# Production of YP170 Vitellogenins Promotes Intestinal Senescence in *Caenorhabditis elegans*

**DOI:** 10.1093/gerona/glz067

**Published:** 2019-03-15

**Authors:** Thanet Sornda, Marina Ezcurra, Carina Kern, Evgeniy R Galimov, Catherine Au, Yila de la Guardia, David Gems

**Affiliations:** 1 Institute of Healthy Ageing, Research Department of Genetics, Evolution and Environment, University College London, UK; 2 Department of Biochemistry, Faculty of Medical Science, Naresuan University, Phitsanulok, Thailand; 3 School of Biosciences, University of Kent, Canterbury, UK; 4 Instituto de Investigaciones Científicas y Servicios de Alta Tecnología, Ciudad del Saber, Panama

**Keywords:** Aging, Age-related pathology, Animal model, *Caenorhabditis elegans*, Yolk

## Abstract

During aging, etiologies of senescence cause multiple pathologies, leading to morbidity and death. To understand aging requires identification of these etiologies. For example, *Caenorhabditis elegans* hermaphrodites consume their own intestinal biomass to support yolk production, which in later life drives intestinal atrophy and ectopic yolk deposition. Yolk proteins (YPs; vitellogenins) exist as three abundant species: YP170, derived from *vit-1*–*vit-5*; and YP115 and YP88, derived from *vit-6*. Here, we show that inhibiting YP170 synthesis leads to a reciprocal increase in YP115/YP88 levels and *vice versa*, an effect involving posttranscriptional mechanisms. Inhibiting YP170 production alone, despite increasing YP115/YP88 synthesis, reduces intestinal atrophy as much as inhibition of all YP synthesis, which increases life span. By contrast, inhibiting YP115/YP88 production alone accelerates intestinal atrophy and reduces life span, an effect that is dependent on increased YP170 production. Thus, despite copious abundance of both YP170 and YP115/YP88, only YP170 production is coupled to intestinal atrophy and shortened life span. In addition, increasing levels of YP115/YP88 but not of YP170 increases resistance to oxidative stress; thus, longevity resulting from reduced vitellogenin synthesis is not attributable to oxidative stress resistance.

The mechanisms underlying the aging process remain poorly defined. According to the hyper-function theory (1–4), one contributory mechanism is futile run-on in later life of developmental and reproductive programs (or quasi-programs), leading to the development of senescent pathologies, including some that limit life span. One way of viewing aging (ie, senescence) is as the set of pathologies that increase in later life (5). This suggests that to understand the causes of aging, including in model organisms such as *Caenorhabditis elegans*, one needs to characterize late-life pathologies and then discover their origins. Where pathologies result from quasi-programs, this requires identifying the biological programs whose run-on generates them.

Aging *C. elegans* exhibits a number of pathologies that are potentially attributable to run-on (6). One such is a steatotic accumulation of yolky lipid, mainly in the body cavity in the form of large oily pools but also in muscles and uterine tumors (7–10). This appears to result from seemingly futile run-on of yolk synthesis in later life. Earlier in adulthood, during hermaphrodite reproduction, developing oocytes are provisioned with yolk, which is lipoprotein that contains several vitellogenin proteins, products of the genes *vit-1*–*vit-6*. Yolk provisioning is assumed to promote larval fitness, although knockdown of yolk production has little effect on progeny production under standard laboratory culture conditions (11). The *C. elegans* vitellogenin (VIT) proteins contain domains homologous to the apoprotein of human low-density lipoprotein, apoB-100 (12,13). In *C. elegans*, yolk proteins (YPs) bind and transport lipids such as triglycerides and cholesterol to oocytes, thereby showing a similar function to low-density lipoprotein in mammals (14). Vitellogenin precursors are synthesized in the intestine and then secreted into the pseudocoelom (body cavity), from which yolk is taken up by developing oocytes by endocytosis *via* the low-density lipoprotein-like receptor RME-2 (15,16).

On electrophoretic gels, four YP bands are visible: the closely running YP170A and YP170B, encoded by *vit-3* to *-5* and *vit-1*, *-2*, respectively, and YP115 and YP88, encoded by *vit-6* (17). *vit-6* initially generates a precursor, YP180, which after leaving the intestine and before being taken up by oocytes is cleaved. *vit-3* and *-4* have more than 99% sequence similarity to each other and 96% sequence similarity to *vit-5*, and *vit-3*, *-4*, *-5* are 68% similar to *vit-1*, *-2*, whereas *vit-6* is 50% similar to the other *vit* genes (17). *vit-1* was at first thought to be a pseudogene, but sequencing of the *C. elegans* genome showed that this is not so (18,19).

*C. elegans* hermaphrodites initially produce sperm but then switch to production of oocytes, which are fertilized by self-sperm. On depletion of self-sperm after 3–4 days, reproduction ceases, but after this vitellogenesis continues and yolk accumulates to high levels in the body cavity (7,8,10,20), with YP170 levels increasing approximately sixfold between day 1 and day 8 of adulthood (10,21). This suggests that *C. elegans* hermaphrodites lack a mechanism to switch off yolk production after sperm depletion, that is, that there exists what is effectively a vitellogenic open faucet (8,10,22).

Yolk synthesis occurs in the intestine, which in *C. elegans* is the main metabolic organ, also performing the functions of liver and adipose tissue, and is a major site of action of genes with effects on life span (23,24). The intestine also exhibits major senescent pathology, most notably severe atrophy, including loss of nuclei and microvilli (10,20). We recently identified a mechanism causing intestinal atrophy, demonstrating that yolk synthesis is coupled to intestinal atrophy, as gut biomass is apparently consumed to sustain yolk synthesis (10). Gut-to-yolk biomass conversion is mediated by autophagy and promoted by insulin/insulin-like growth factor 1 (IGF-1) signaling, and several interventions that reduce intestinal atrophy also increase life span (10).

In this study, we verify the vitellogenic open faucet model, showing that yolk accumulation results from a relatively steady flow of continued synthesis of yolk during adulthood combined with cessation of egg laying. We also describe how blocking YP115/YP88 synthesis increases YP170 levels, which accelerates gut atrophy and shortens life span; thus, it is YP170 production specifically that is a major driver of *C. elegans* senescence. In addition, increased YP115/YP88 level protects against oxidative stress but does not increase life span, suggesting that life span is not limited by oxidative stress. These findings are broadly consistent with the hyper-function theory (1–4).

## Methods

### Culture Methods and Strains

*Caenorhabditis elegans* were maintained using standard conditions (25), at 20°C on nematode growth medium (NGM) plates seeded with *Escherichia coli* OP50, or HT115 for RNA interference (RNAi), performed as described previously (26). RNAi trials were initiated from egg hatching, unless otherwise stated. The following strains were used: N2 hermaphrodite stock (27), DH26 *rrf-3(b26) II* (formerly *fer-15*), EG3234 *oxIs144 [inx-16p::inx-16::GFP, lin-15+],* GA504 *wuIs54 [pPD95.77 sod-1p::sod-1::GFP, rol-6(su1006)]* (28), GA631 *wuIs177 [ftn-1p::GFP, lin-15(+)]* (29), JK574 *fog-2(q71) V*, and LD1171 *ldIs3 [gcs-1p::GFP + rol-6(su1006)]*.

### Mating Protocol

*fog-2(q71)* or *rrf-3(b26)* males were used for mating tests. Animals were picked at the L4 stage and added to NGM plates at a ratio of 3:1 males to hermaphrodites or females and left to mature and mate for 24 hours, after which males were removed. *rrf-3* males were raised and mated at 25°C, the nonpermissive temperature for the *rrf-3(b26)* fertilization defective (Fer) phenotype; after removal of *rrf-3* males, *fog-2* females were shifted to 20°C.

### Nematode Protein Content Measurements

Total protein was measured using bicinchoninic acid. One hundred worms were harvested into 50 μL of M9 buffer and frozen at –80°C until used. Samples were added with 250 μL of CelLytic M buffer (Sigma-Aldrich) containing 1:1,000 protease inhibitor cocktails, sonicated with a Bioruptor (Cosmo Bio Co., Ltd) for 8 minutes with 30-second intervals and centrifuged at 4°C at 6,000 rpm for 15 minutes. The bicinchoninic acid procedure was performed using 96-well plates. For each well, 200 μL testing solution was mixed with 25 μL sample or bovine serum albumin standards. The plate was mixed gently, incubated at room temperature for 2 minutes, and incubated at 37°C for 30 minutes. The plate was measured for absorbance at 620 nm.

### YP Measurements

Yolk levels were quantified by Coomassie blue staining. Twenty worms were harvested into 25 μL of M9 buffer and frozen at –80°C until used. Samples were added with 25 μL of 2× Laemmli sample buffer (Sigma-Aldrich), incubated at 70°C and vortexed periodically for 15 minutes, and then incubated at 95°C for 5 minutes and centrifuged at 6,000 rpm for 15 minutes. Sodium dodecyl sulfate–polyacrylamide gel electrophoresis (SDS-PAGE) was performed, using Criterion XT Precast Gels 4–12% Bis-Tris (Bio-Rad) and XT MOPS (Bio-Rad) as a running buffer. Gels were stained with Coomassie blue following standard protocols. Gels were analyzed using ImageQuant LAS 4000 (GE Healthcare). Protein band identification was based on published data (21). YPs were normalized to myosin and the ratio of actin to myosin was used to give an indication of the reliability of myosin as a standard.

### Microscopy

Worms were mounted onto 2% agar pads and anesthetized with 0.2% levamisole, and coverslips were gently placed onto the pads. Nomarski images of worms were acquired using a Zeiss microscope with a Hamamatsu ORCA-ER digital camera C4742-95 using Volocity software, version 6.3 (Improvision, Perkin-Elmer). For fluorescence images, a fluorescein isothiocyanate (FITC)/green fluorescent protein (GFP) filter cube (excitation range 450–490 nm, emission range 515–565 nm) was used. A constant exposure time was maintained between samples in fluorescence intensity comparisons.

### Intestinal Atrophy and Yolk Pool Accumulation Measurements

Intestinal atrophy and yolk pool accumulation were measured as described previously, using Nomarski microscopy (10). Intestinal atrophy was quantified by measuring the intestinal width at the mid-part of the posterior intestine, subtracting the lumen width and dividing by the body width (10). Yolk pool accumulation was quantified by measuring total yolk pool area and dividing by body area (10).

### RNA Isolation and Quantitative Polymerase Chain Reaction

Approximately 150 worms were collected in 50 μL of M9, to which 800 μL Trizol (Sigma-Aldrich) was rapidly added, and samples were then stored at –80°C until processing. Complementary DNA was synthesized using the SuperScript IV Reverse Transcriptase (Invitrogen). SYBR Green Real-Time PCR was performed using a QuantStudio 6 Flex Real-Time PCR System (Thermo Fisher Scientific) and normalized using the ΔΔCt method as described previously (30).

### Life-span Assays

Animals were maintained at 20–25 worms per plate. Life spans were scored every day or every other day for dead worms, starting from day 1 of adulthood. Raw mortality data for all trials are provided (Supplementary Dataset 2).

### Oxidative Stress Resistance Assays

L4 stage worms were transferred to plates containing 40 mM paraquat (Sigma-Aldrich) or 7.5 mM *tert*-butyl hydroperoxide (*t*-BOOH; Sigma-Aldrich). These plates had been seeded with HT115 bearing RNAi plasmids for 2 days. Animals were incubated at 20°C and, from day 1 of adulthood, scored for survival daily or hourly for paraquat or *t*-BOOH, respectively. Raw mortality data for all trials are provided (Supplementary Dataset 2).

### Statistical Analysis

The Student’s *t* test was used. One-way analysis of variance was performed for multiple comparisons, and Tukey–Kramer multiple comparison was used to obtain statistical comparisons between all groups. For survival assays, statistical significance was estimated using log-rank and Wilcoxon tests using JMP software, version 13.0 (SAS Institute, Inc.). Graphs display mean values and all error bars depict standard error of the mean.

## Results

### A Vitellogenic Open Faucet Contributes to Yolk Accumulation

**Figure 1.**
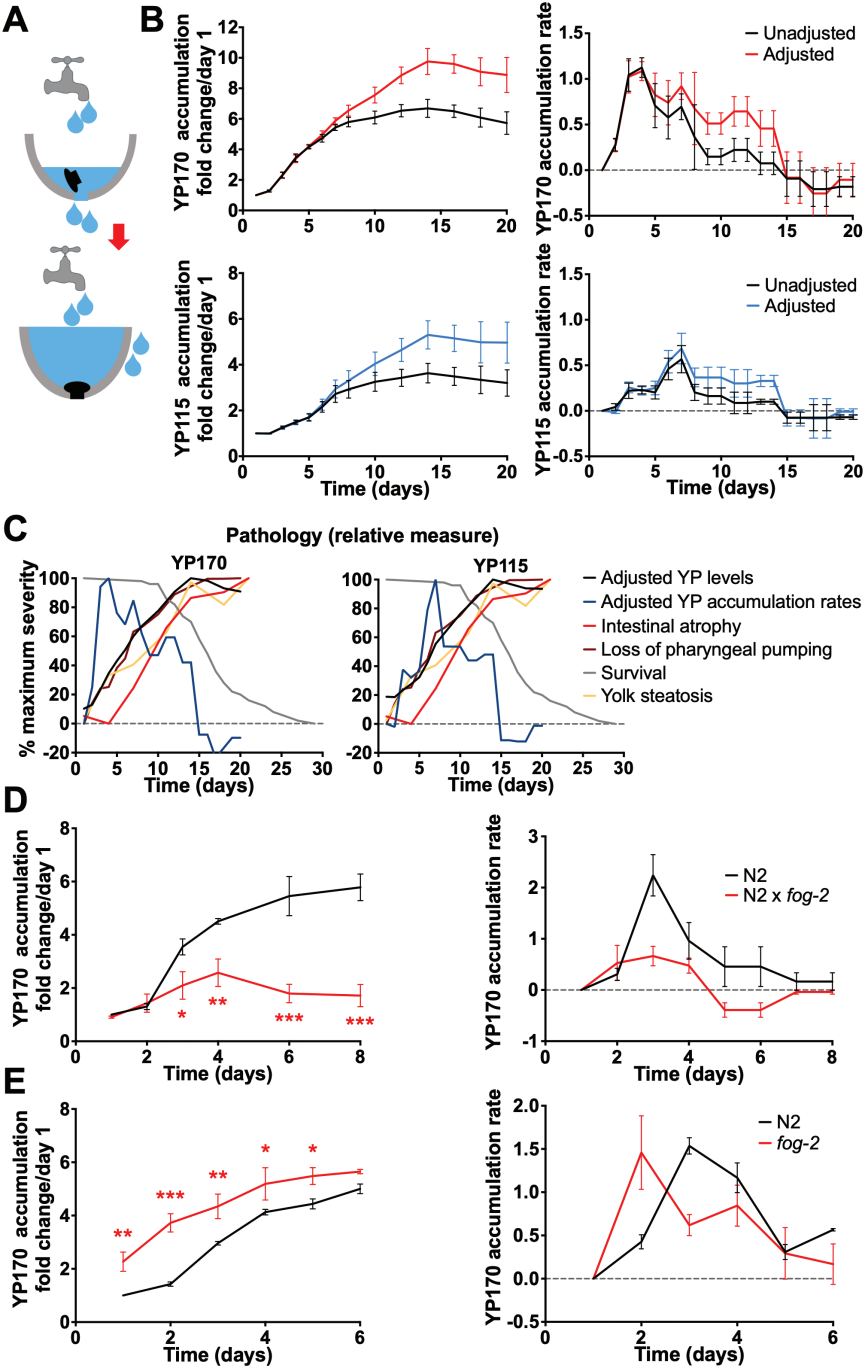
Yolk accumulation results from continued vitellogenesis and cessation of egg laying. (**A**) Cartoon showing faucet and sink model. (**B**) Age changes in vitellogenin accumulation (left) and accumulation rate (right), summed data from four trials. Top, YP170; bottom, YP115. Data shown with and without adjustment for age changes in intestinal volume. (**C**) Combined figure showing yolk protein (YP) adjusted accumulation and accumulation rate, and other age changes. Left, with YP170; right, with YP115. (**D**) Mating of N2 hermaphrodites with males (*fog-2*) reduces YP170 accumulation. Left, total accumulation. Right, accumulation rate. (**E**) YP170 accumulation in spermless *fog-2(q71)* females. Left, total accumulation. Right, accumulation rate. (D and E) Summed data from three trials, age-matched comparison. Data are mean ± SEM, *p < .05, **p < .01, ***p < .001.

A working hypothesis is that continued vitellogenesis after sperm depletion promotes visceral pathology in *C. elegans* hermaphrodites. According to this view, yolk accumulation is a function of (a) unabated yolk production in adult hermaphrodites, and (b) a decline in egg laying. This model is analogous to an open faucet filling a sink: the content of the sink reflects the rate of flow of the faucet (cf. yolk synthesis) and the presence or absence of an open plug hole (c.f. egg laying; Figure 1A).

An open faucet mechanism predicts a steady rate of YP accumulation after the cessation of egg laying. To test this, we examined how the rate of vitellogenin accumulation changes with increasing age in selfed wild-type hermaphrodites, by reanalyzing previously reported YP accumulation data (10). Age changes in yolk accumulation rate could reflect changes in yolk production rate per unit mass of intestine and/or changes in intestinal biomass. The hermaphrodite intestine increases in size from day 1 to day 4 as the adult hermaphrodite grows and then decreases due to gut atrophy (10). We, therefore, adjusted YP accumulation rate data for gut volume changes. Relative intestinal volume from day 1 to day 20 was estimated from the diameters of the intestine and intestinal lumen, taking the intestine to be a hollow cylinder, and using data for aging N2 hermaphrodites from our previous study (10). For YP170 and YP115 adjusted accumulation rate, this revealed an early peak followed by an extended period of relatively steady accumulation (Figure 1B; Supplementary Figure 1 [representative SDS-PAGE gel and individual trials]; Supplementary Dataset 1 [raw data]). This supports the presence of open faucet-type yolk synthesis. Peak accumulation rate was earlier for YP170 than YP115, occurring on day 4 and day 7, respectively, implying a difference in VIT-6 synthesis and/or trafficking. For both YP170 and YP115, accumulation ceases abruptly on day 15, followed by a phase of apparent decline in YP levels.

To put these findings into context, adjusted YP170 and YP115 accumulation rate changes were plotted against other parameters of senescence: cumulative YP accumulation, declining pharyngeal pumping rate, intestinal atrophy, and survival (life span; Figure 1C). This shows that YP accumulation is still occurring when the first animals are dying from senescence, and ceases shortly after gut atrophy reaches its peak level. It also shows an approximate correspondence between decline in both the YP accumulation rate and pharyngeal pumping (ie, feeding) rate, possibly due to reduced uptake of food to support yolk biosynthesis. Also notable is the lag between YP accumulation and intestinal atrophy, which is closely correlated with pseudocoelomic lipoprotein pool (PLP; ie, yolk pool) accumulation (10). This could indicate that gut-to-yolk biomass conversion largely contributes to the later stages of yolk accumulation; a further implication is that the growth of the PLPs occurs particularly during the later stages of intestinal atrophy, possibly due to release of intestinal lipid depots (10).

### Cessation of Egg Laying Contributes to Yolk Accumulation

One possible expectation in the source-sink model (Figure 1A) is that during the egg-laying phase, the yolk production rate is set to approximately match the requirements of egg production, and therefore that YP accumulation rate should only rise substantially after days 4–5 (20°C) and sperm depletion. This was true for YP115 but not YP170, whose accumulation rate peaked earlier, on days 3–4 (Figure 1B). This could imply that rate of synthesis of YP170 but not VIT-6 exceeds requirements of egg production.

Another prediction of the source-sink model is that extending the egg-laying period will postpone yolk accumulation whereas blocking egg production will bring it forward. As predicted, extending the egg-laying period by mating with males largely prevented YP170 accumulation (Figure 1D; Supplementary Figure 2), consistent with previous observations of PLPs (10). However, on days 2–4 a slight accumulation of YP was seen, consistent with a slight biosynthetic surplus; mating was previously shown to increase *vit* messenger RNA (mRNA) levels, and this could be a contributory factor here (21).

The drop in YP accumulation rate after reproduction (Figure 1B) could imply that the presence of sperm increases YP production rate. However, an early peak in YP170 accumulation rate was also seen in spermless *fog-2(q71)* mutant females (Figure 1E; Supplementary Figure 3), although YP170 initially accumulated faster, probably due to the absence of egg laying in *fog-2(q71)* females; this is consistent with earlier increases in YP accumulation previously noted in sterile *fem-1(hc17)* and *fem-3(q20gf)* mutant hermaphrodites (21). Similarly, blocking yolk transport into oocytes using *rme-2* RNAi accelerated YP accumulation (Supplementary Figure 4). Moreover, mating *fog-2* females with *rrf-3(b26)* (formerly *fer-15*) males, which can mate but which produce fertilization-defective sperm, did not alter yolk accumulation rate (Supplementary Figure 5). Again, mating *fog-2* females with *fog-2* males largely prevented YP accumulation (Supplementary Figure 6).

These results verify that YP accumulation pattern is a function of both YP synthesis and YP disposal by egg laying and confirm that postreproductive hermaphrodites maintain a steady rate of YP accumulation into late adulthood.

### Knockdown of YP170 Synthesis Increases YP115/YP88 Levels and Vice Versa

**Figure 2.**
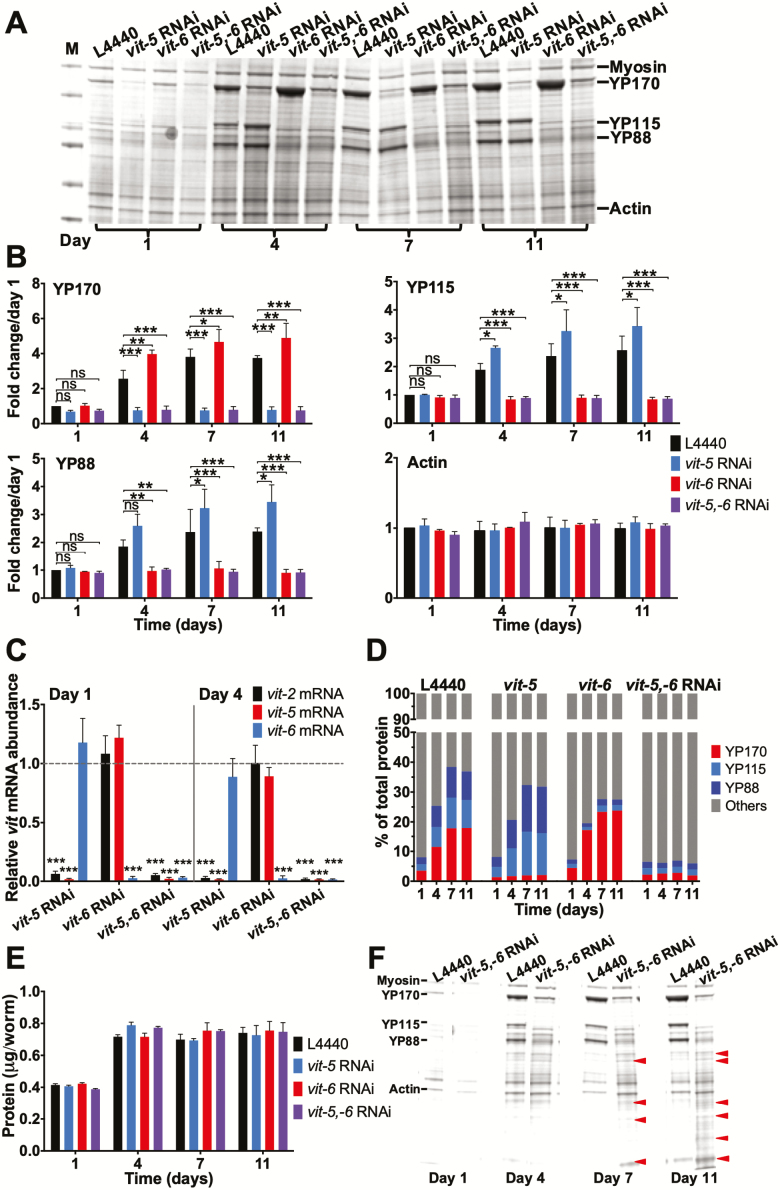
Knockdown of YP170 synthesis reciprocally increases YP115/YP88 levels and *vice versa*. (**A**) Coomassie gel showing the effects of *vit-5* and/or *vit-6* RNAi on yolk protein (YP) levels. (**B**) Quantified data, fold change in YP levels. Note that *vit-5* RNAi increases levels of YP115 and YP88, whereas *vit-6* RNAi increases YP170 accumulation. Statistical comparisons are to L4440 control on the same day. (**C**) Effect of *vit-5* and/or *vit-6* RNAi on *vit-2*, *vit-5* or *vit-6* mRNA levels, compared to L4440 control on the same day. Note that *vit-5* RNAi does not increase *vit-6* mRNA levels and *vit-6* RNAi does not increase *vit-5* mRNA levels. (**D**) No change in the YP content per worm after *vit-5* or *vit-6* RNAi. (**E**) No effect of *vit* RNAi on total protein content. (**F**) Appearance of new protein bands after *vit-5,-6* RNAi. All data are mean ± SEM, *p < 0.05, **p < 0.01, ***p < 0.001.

During *C. elegans* aging, continued yolk production is coupled to intestinal atrophy, and blocking vitellogenin synthesis inhibits intestinal atrophy (10). But is this due to the synthesis of particular vitellogenins or overall vitellogenin synthesis? Combined *vit-5 + vit-6* RNAi is sufficient to largely prevent accumulation of all YP species (10). We found that *vit-5* RNAi alone largely blocked YP170 accumulation (Figure 2A and B); given the close sequence similarity between *vit-3, -4*, and *-5* (17), it is likely that expression of all three genes is abrogated. *vit-5* RNAi also greatly reduces *vit-2* mRNA levels (Figure 2C). Unexpectedly, *vit-5* RNAi also increased YP115 and YP88 accumulation by up to 44% (Figure 2A and B). Similarly, *vit-6* RNAi blocked YP115 and YP88 accumulation but increased YP170 accumulation by up to 55% (Figure 2A and B).

*vit-5* RNAi had little effect on *vit-6* mRNA and *vice versa* (Figure 2C). Thus, this reciprocal effect does not involve a mechanism affecting *vit* gene transcription. This suggests that reducing the abundance of a given *vit* mRNA species leads to increased translation of other *vit* mRNA species, perhaps reflecting competition between *vit* mRNAs for access to translational machinery.

*C. elegans* hermaphrodites produce copious amounts of yolk, such that as a proportion of total YP levels rise from less than 10% on day 1 of adulthood to almost 40% by day 11 (Figure 2D) (10). As expected, this increase was entirely suppressed by *vit-5,-6* RNAi, but due to reciprocal increases in synthesis, *vit-5* or *vit-6* RNAi alone only slightly reduced overall YP content (Figure 2D). This suggests that *vit-5,-6* double RNAi but not *vit-5* or *vit-6* RNAi should reduce total worm protein content. But surprisingly, not even *vit-5,-6* RNAi reduced overall protein content (Figure 2E). This implies that other proteins are present in place of vitellogenin after *vit-5,-6* RNAi.

To explore this further, we compared Coomassie-stained protein gels of extracts from control and *vit-5,-6* RNAi-treated N2 hermaphrodites. This revealed, in the latter, the presence of numerous bands showing an increased abundance of proteins other than vitellogenin (Figure 2F).

### Vitellogenin Synthesis Reduces Levels of Other Intestinal Proteins

**Figure 3.**
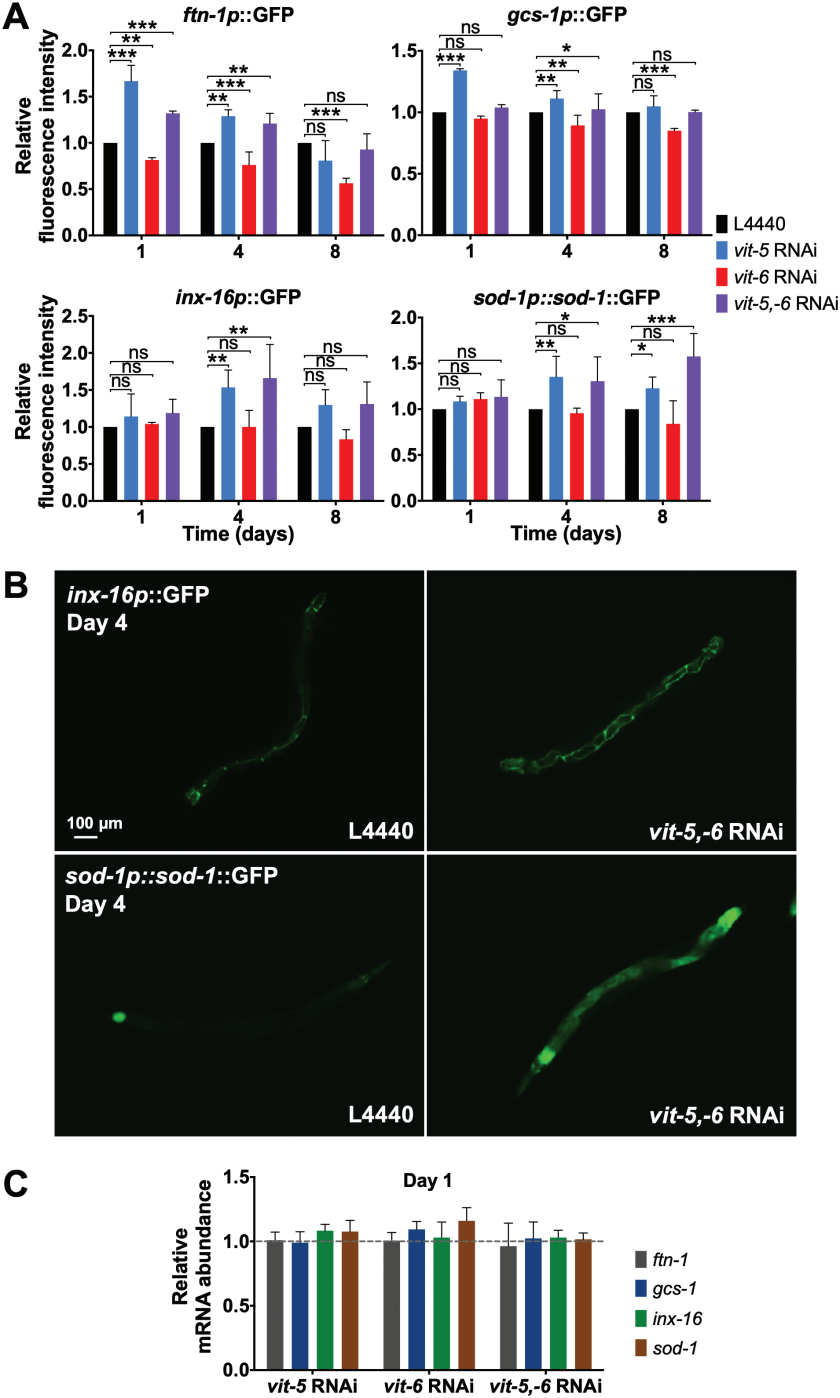
Vitellogenin synthesis reduces levels of other intestinal proteins. (**A**) Effects of *vit* RNAi on selected fluorescent intestinal reporters, compared to L4440 control on same day, data are mean ± SEM, *p < 0.05, **p < 0.01, ***p < 0.001. (**B**) Selected examples of effects of *vit-5,-6* RNAi on intestinal reporter gene expression. Scale bars, 100 μm. (**C**) Effects of *vit* RNAi on mRNA for selected intestinal genes, compared to L4440 control on the same day; data are mean ± SEM.

How does *vit-5,-6* RNAi increase levels of other proteins? One possibility is that knockdown of all YPs increases synthesis of other intestinal proteins, perhaps due to the absence of monopolization of the protein synthesis machinery in the hermaphrodite intestine by *vit* mRNAs. To probe this, we tested effects of *vit-5, vit-6*, or *vit-5,-6* RNAi on selected fluorescent reporters of intestinally expressed genes (*ftn-1, gcs-1, inx-16*, and *sod-1*) on days 1, 4, and 8 of adulthood. The prediction here was that *vit-5,-6* RNAi but not *vit-5* or *vit-6* RNAi alone would increase reporter expression. However, against expectation, both *vit-5* and *vit-5,-6* RNAi consistently increased expression of all reporters, whereas *vit-6* RNAi reduced fluorescence (Figure 3A and B). *vit-5,-6* RNAi did not alter *ftn-1, gcs-1, inx-16* or *sod-1* mRNA levels (day 1; Figure 3C) supporting the view that vitellogenin synthesis affects levels of other proteins by posttranscriptional mechanisms.

That both *vit-5* and *vit-5,-6* RNAi, with elevated and reduced levels of YP115/YP88, respectively, similarly increase intestinal reporter expression, suggests that the synthesis of YP170 but not YP115/YP88 reduces levels of other intestinal proteins.

### *vit-6* RNAi Enhances Intestinal Atrophy and Shortens Life Span

**Figure 4.**
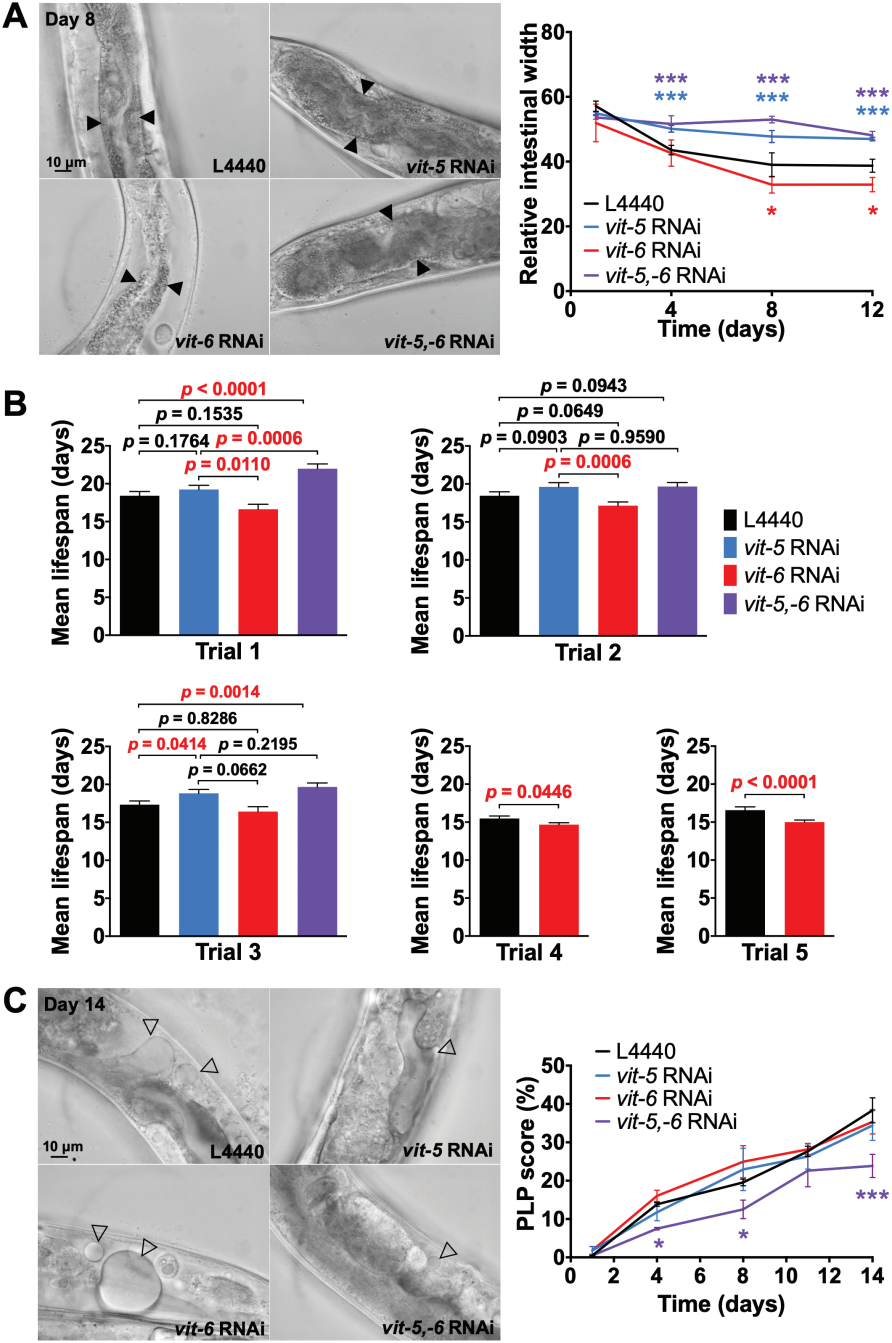
YP170 production accelerates intestinal atrophy and shortens life span. (**A**) Effect of *vit* RNAi on intestinal atrophy. Left, microscope images, scale bars, 10 μm. Right, quantitation. (**B**) Effect of *vit* RNAi on life span. Bar graphs depicting mean ± SEM of life spans for individual trials; *p*, log-rank test. (**C**) Effect of *vit* RNAi on yolk pool accumulation. Left, microscope images, scale bars, 10 μm. Right, quantitation (A and C). Filled arrowheads, intestine. Open arrowheads, PLPs. Data are mean ± SEM, age-matched comparison, *p < 0.05, ***p < 0.001.

Abrogation of yolk accumulation by *vit-5,-6* RNAi inhibits intestinal atrophy and extends life span (10). Next, we tested the effects of *vit-5* or *vit-6* RNAi alone on these traits (20°C). *vit-5* RNAi, which increases YP115/YP88 levels, proved to suppress intestinal atrophy to a similar extent as *vit-5,-6* RNAi (Figure 4A). *vit-5* RNAi caused a modest increase in life span that was statistically significant in 1/3 trials (Figure 4B; Supplementary Table 1; Supplementary Dataset 2), consistent with previous observations (31). Furthermore, *vit-5,-6* RNAi more robustly increased life span, both relative to RNAi controls (L4440), as observed previously (10), and to *vit-5* RNAi alone (statistically significant in 2/3 trials in each case; Figure 4B; Supplementary Table 1).

Interestingly, *vit-6* RNAi, which increases YP170 levels, both enhanced intestinal atrophy (Figure 4A) and reduced life span (Figure 4B; Supplementary Figure 7; Supplementary Table 1). In five trials, mean life span estimates with *vit-6* RNAi were lower than controls, and in 2/5 cases the effect was statistically significant, suggesting a modest but real effect on life span. Notably, *vit-5* RNAi suppresses the life-shortening effects of *vit-6* RNAi on life span (Figure 4B; Supplementary Table 1). Taken together, this implies that production of YP170 but not YP115/YP88 promotes intestinal atrophy and reduces life span (see Discussion section).

*vit-5,-6* RNAi also inhibits PLP accumulation (10). However, neither *vit-5* RNAi or *vit-6* RNAi alone had a detectable effect on PLP accumulation (Figure 4C), suggesting that high levels of either YP170 or YP115/YP88 are sufficient to assure normal levels of yolk lipid production and/or transport into the body cavity.

### Increased YP115/YP88 Levels Are Associated With Oxidative Stress Resistance

**Figure 5.**
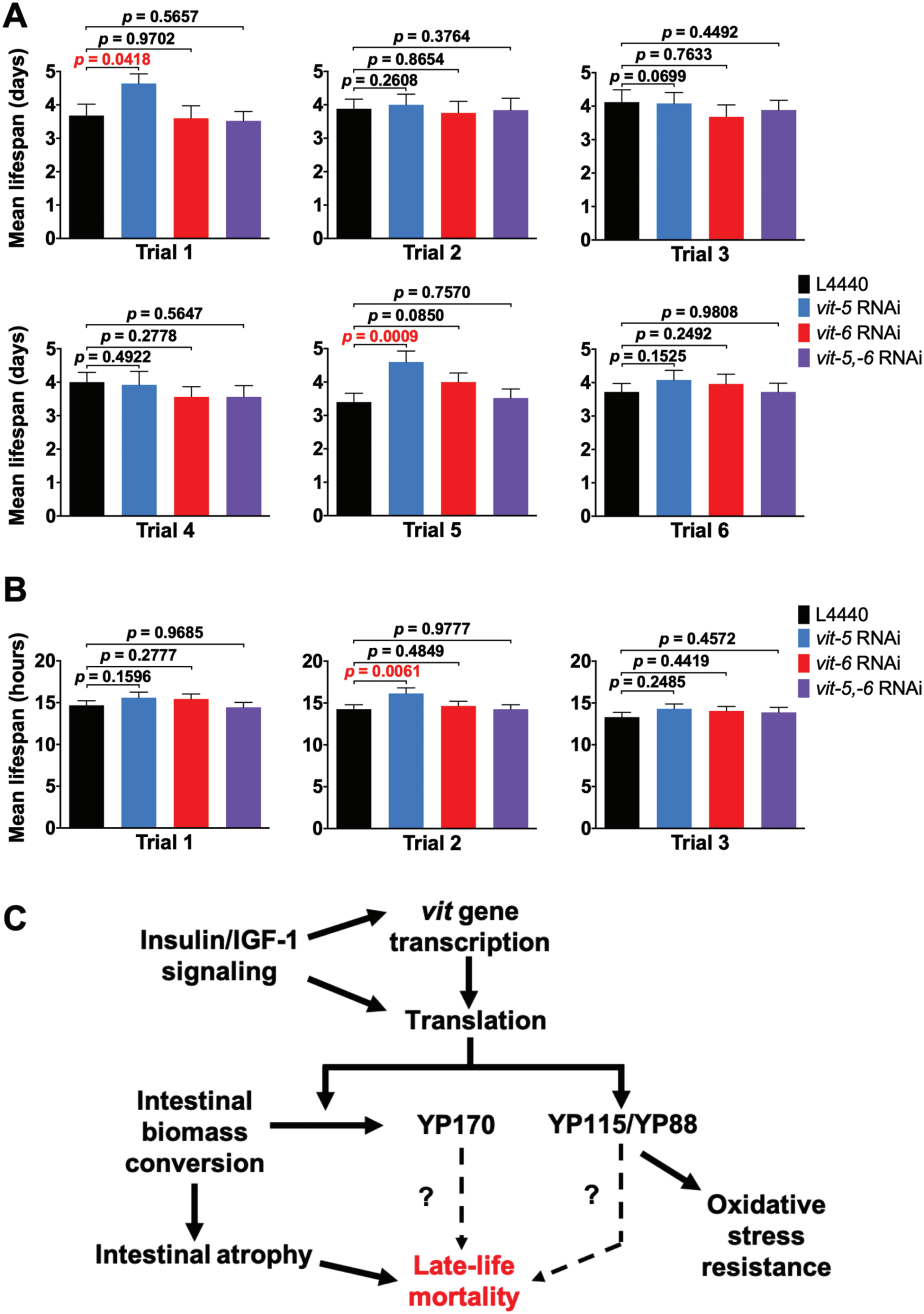
Evidence that increased YP115/YP88 can enhance oxidative stress resistance. (**A** and **B**) Effect of *vit* RNAi on resistance to oxidative stress caused by 40 mM paraquat (A) and 7.5 mM *t*-BOOH (B); bar graphs depicting mean survival ± SEM for individual trials; *p*, log-rank test. (**C**) Model for distinct roles of YP170 and YP115/YP88 in *C. elegans* senescence. Rate of intestinal atrophy is coupled to synthesis of YP170 but not YP115/YP88, through gut-to-yolk biomass conversion (10). Higher YP170 levels reduce life span because production is coupled to intestinal atrophy; but effects of increased YP170 cannot be ruled out. Blocking both YP170 and YP115/YP88 synthesis increases life span more than YP170 alone, suggesting that YP115/YP88 accumulation could contribute to late-life mortality. Wild-type insulin/IGF-1 signaling greatly shortens life span (35,36), increases *vit* gene transcription (31,37) and translation (21), and global translation (38,39), and intestinal atrophy (10).

In aging *C. elegans*, elevated protein oxidation levels were observed in YP115 (but not YP88) raising the possibility that YP115 has antioxidant properties (32). This is consistent with reported antioxidant properties of vitellogenin in honeybee workers (33). To test this further, we tested effects on resistance to oxidative stress (Oxr), using either 40 mM paraquat, or 7.5 mM *t*-BOOH, of RNAi of either *vit-5* or *vit-6,* or both. In no cases was a reduction in Oxr observed, but *vit-5* RNAi, which increases YP115/YP88 levels, increased Oxr in a number of trials (statistically significant in 2/6 trials for paraquat and 1/3 for *t*-BOOH; Figure 5A and B; Supplementary Figure 8 ; Supplementary Tables 2 and 3). That *vit-5,-6* RNAi did not increase Oxr implies that this effect of *vit-5* RNAi is attributable to increased YP115/YP88 rather than reduced YP170. This suggests that YP115 and/or YP88 but not YP170 can protect against oxidative stress.

## Discussion

This study adds more detail to our understanding of the causes of yolk steatosis in aging *C. elegans*, and how yolk production promotes intestinal senescence. It verifies the presence of a vitellogenic open faucet in post-reproductive hermaphrodites and reveals that production of YP170 but not YP115/YP88 is coupled to intestinal atrophy, implying that gut-to-yolk biomass conversion specifically involves YP170. Thus, YP170 production specifically promotes senescent pathogenesis in *C. elegans*. More broadly, these results support the view that system run-on, rather than system failure or stochastic damage accumulation, causes senescent intestinal atrophy and yolk steatosis.

### Yolk Accumulation Results From Continued Synthesis and Cessation of Egg Laying

It has long been known that yolk accumulates in *C. elegans* hermaphrodites after sperm depletion in *C. elegans* (7,8,21). Here, a likely candidate mechanism is loss of yolk efflux through egg laying combined with continued yolk production. Our findings confirm the presence of such a source and sink mechanism and also reveal a number of additional details. First, there is an early peak of YP170 accumulation before sperm depletion (Figure 1B and C), suggesting that yolk production exceeds demand during reproduction, at least under replete nutritional conditions. Second, this early peak of yolk production is independent of the presence of sperm, as shown by its occurrence in spermless *fog-2* females (Figure 1E). Third, YP115/YP88 levels peak later than those of YP170, suggesting a difference in the synthesis and/or trafficking of VIT-6 (Figure 1B,C). Fourth, there is a relatively steady rate of yolk accumulation in post-reproductive adults, consistent with an unregulated, open faucet mechanism, which ceases abruptly on day 15, perhaps as the result of further intestinal senescence and incipient death (Figure 1B).

We conclude that PLP accumulation, or senescent yolk steatosis, is caused by a combination of post-reproductive continuation of yolk synthesis, and sperm depletion that is a consequence of the protandrous architecture (or bauplan) (34) of the *C. elegans* hermaphrodite germline (ie, because it makes sperm first and then oocytes).

### YP170 Production Drives Intestinal Atrophy and Shortens Life Span

New features of the mechanisms of senescent pathogenesis in *C. elegans* may be deduced from the effects of selective inhibition of YP170 or YP115/YP88, as follows. Combined *vit-5,-6* RNAi suppressed gut atrophy and increased life span to a similar degree to *vit-5* RNAi, despite elevated YP115/YP88 levels in the latter (Figure 5C). By contrast, *vit-6* RNAi, which increases YP170 levels, increased gut atrophy but not if the increase in YP170 was abrogated by simultaneous *vit-5* RNAi (Figure 5C). Taken together, this implies that YP170 production or abundance drives intestinal atrophy but that of YP115/YP88 does not. This suggests the presence of a senescence-promoting mechanism that specifically couples YP170 synthesis to disappearance of intestinal cell contents. Similarly, effects of RNAi on life span indicate a predominant role of YP170 in life span determination. Moreover, that *vit-6* RNAi both accelerates gut atrophy and shortens life span provides further evidence that senescent atrophy of the intestine contributes to late-life mortality.

### Possible Causes of Reciprocal Changes in YP Species After *vit* Gene RNAi

RNAi knockdown of YP170 caused a reciprocal increase in YP115/YP88 levels and *vice versa*. This could imply that the intestinal protein biosynthetic machinery is working at full capacity to produce yolk, so that *vit* mRNAs compete with one another for access to ribosomes. Possible reasons for this are hyperabundance of *vit* mRNAs and preferential access of *vit* mRNAs to the translational machinery. We explored both possibilities by the analysis of published RNA sequencing (RNA-seq) and ribosome profiling (ribo-seq) data from a study of whole-worm mRNA extracts from young adult hermaphrodites (40). Examining RNA-seq data, *vit-6* mRNA was the third most abundant (4,981 reads per kilobase million (RPKM); Supplementary Table 4), and this was exceeded by the sum of mRNAs encoding YP170 (*vit-1–5*, 9,628 RPKM; Supplementary Table 5). Given that these values are for whole-worm extracts, this implies that *vit* mRNAs are very abundant indeed within the intestine.

As an indicator of possible preferential translation of *vit* mRNAs, we tested for high representation of *vit* mRNA in ribo-seq profiles relative to RNA-seq profiles. To do this, we calculated the ratio of mRNA abundance in ribo-seq versus mRNA seq data and compared the mean values for the 4 *vit* mRNAs for which data were available with that for the top 20 other most abundant mRNAs (RNA-seq data). This gave values of 1.15 and 1.63, respectively, consistent with possible underrepresentation of *vit* mRNAs on ribosomes (Supplementary Table 5), although if one excludes the three most abundant ribo-seq mRNAs from this analysis (*col-140*, *eef-1A.1*, and *rps-7*) the latter value is reduced to 1.18. The combination of very high *vit* mRNA abundance in RNA-seq profiles and possible underrepresentation in ribo-seq data suggests that high vitellogenin translation levels are caused by high *vit* mRNA abundance and that *vit* mRNA hyperabundance leads to competition between *vit* mRNA species for access to ribosomes.

However, this scenario does not rule out the presence of other mechanisms enhancing *vit* mRNA translation rate, whose presence could be masked by very high *vit* mRNA levels. Preferential access of *vit* mRNAs to the translational machinery could occur due to mRNA sequence features or by suppression of non-*vit* mRNA translation. During some forms of viral infection, 5′-cap-dependent translation is suppressed and viral mRNAs are translated *via* internal ribosome entry site (IRES) sequences (41). However, examination of *vit* mRNA sequences using the RNAfold web server (42,43) did not reveal any potential IRES sequences. Notably, though, none of the *vit* mRNAs bear spliced leader sequences (WormBase;www.wormbase.org, release WS267, 2018). One possibility is that the absence of a requirement for *trans*-splicing enables faster production of mature *vit* mRNA species.

One hypothesis that we entertained is that translation of *vit* mRNAs competes with translation of other intestinal proteins, which contributes to intestinal atrophy. Our tests showed that both *vit-5* and *vit-5,-6* RNAi increased levels of intestinal reporter gene proteins but not their corresponding endogenous mRNAs (Figure 3). This could imply that synthesis of YP170 inhibits translation of other proteins. However, increased reporters could reflect the fact that *vit-5* and *vit-5,-6* RNAi inhibit gut atrophy (Figure 4A), although increased reporter levels even on day 1 (Figure 3A), long before atrophy appears, argues against this.

Taken together, these results favor the view that high *vit* mRNA abundance rather than preferential translation mechanisms leads to competition with other mRNAs for access to translational machinery. However, they also suggest a weaker competitive effect of *vit-6* mRNA (given that YP170 synthesis specifically is coupled to intestinal atrophy).

### Effects of Vitellogenins on Oxidative Stress Resistance and Life Span

*vit-5* RNAi increases YP115/YP88 levels and causes Oxr, and both effects are suppressed by *vit-6* RNAi (Figure 2A and B; Figure 5A and B). This is consistent with a previously suggested antioxidant role for YP115 (32). We previously described an age increase in Oxr (*t*-BOOH) in N2 hermaphrodites (44); one possibility is that VIT-6 accumulation contributes to this age increase in Oxr.

The early, influential theory that reactive oxygen species are a major cause of aging (45,46) suggests that vitellogenins should protect against aging. In fact, knockdown of vitellogenin expression can increase life span in *C. elegans* (10,31) (this study) and in the lubber grasshopper, *Romalea microptera* (47), but reduce it in the honeybee, *Apis mellifera* (48). Here, we show that nematodes subjected to *vit-6* RNAi are Oxr but shorter-lived than non-Oxr *vit-5,-6* and *vit-5* RNAi populations (Figure 4B; Figure 5A and B), that is, effects of vitellogenins on Oxr and longevity can be uncoupled. This is consistent with numerous findings arguing against the view that reactive oxygen species are a major cause of senescence in *C. elegans* (28,49,50). However, it remains possible that reactive oxygen species damage contributes to some senescent pathologies that do not limit *C. elegans* life span under standard culture conditions.

### Later Vitellogenesis: Alternative Models of Antagonistic Pleiotropy

Why after sperm depletion do *C. elegans* continue to synthesize yolk, even causing intestinal pathology to do this? We suggest two possibilities, both consistent with the evolutionary principle of antagonistic pleiotropy. This principle postulates that genes can exert a variety of effects on phenotype throughout life and that earlier effects will have greater impact on fitness due to smaller relative reproductive output at later ages. Consequently, selection may favor alleles with greater early-life fitness benefits even if they cause pathology in later life (ie, senescence) (1).

The first possibility is that continued yolk production represents nonadaptive run-on of processes that contribute to fitness in earlier life (8). The absence of a mechanism to turn yolk production off could reflect the lack of any selective advantage that such an off switch would confer. Such a mechanism is consistent with Blagosklonny’s quasi-program model of antagonistic pleiotropy action, where late-life action of wild-type genes directly promote pathogenetic biological programs (rather than indirectly through effects on damage accumulation) (1,2). Quasi-programs are also a major cause of uterine tumor formation in aging *C. elegans* (4).

A second possibility is that continued yolk production contributes to fitness by some mechanisms as yet unidentified, for example, by increasing reproductive success after later-life mating. Such a mechanism would represent a direct reproductive cost comparable, for example, to costs of lactation in female mammals, which include bone atrophy due to release of bone calcium for milk production (51). Either way, understanding the mechanisms involved should be informative with respect to the identification of general principles of senescent pathophysiology.

## Funding

This work was supported by el Instituto para la Formación y Aprovechamiento de Recursos Humanos, y la Secretaria Nacional de Ciencia, Tecnología e Innovación, Panamá (Y.G.), Naresuan University, Thailand (T.S.), and a Wellcome Trust Award (098565/Z/12/Z to D.G.).

## Supplementary Material

glz067_suppl_Supplementary_Dataset_1

glz067_suppl_Supplementary_Dataset_2

glz067_suppl_Supplementary_Material
