# Supplementary material for: Production of YP170 Vitellogenins Promotes Intestinal Senescence in Caenorhabditis elegans
Source: J Gerontol A Biol Sci Med Sci. 2019 Mar 15;74(8):1180–8. doi: 10.1093/gerona/glz067 (PMC6625598; doi:10.1093/gerona/glz067)
Supplement: glz067_suppl_Supplementary_Dataset_1 [file glz067_suppl_supplementary_dataset_1.docx]

**Supplementary Dataset 1**. Raw YP measurement data.

**A.** cf. Figure 1B; Supplementary Figure 1.

| Days | Bands | N2 | | | |
| --- | --- | --- | --- | --- | --- |
|  |  | Trial 1 | Trial 2 | Trial 3 | Trial 4 |
| 1 | Myosin | 779970 | 1948629 | 1072111 | 4307796 |
|  | YP170 | 831761 | 1927249 | 1100543 | 4204234 |
|  | YP115 | 720655 | 1651082 | 1056987 | 3852565 |
|  | YP88 | 831007 | 1801685 | 1129541 | 3909079 |
|  | Actin | 1301560 | 1923362 | 1376596 | 3357942 |
| 2 | Myosin | 1647679 | 2469413 | 927985 | 4865907 |
|  | YP170 | 2017465 | 3409586 | 1375094 | 5619465 |
|  | YP115 | 1502438 | 2449379 | 929334 | 4143823 |
|  | YP88 | 1664906 | 2786367 | 995317 | 4335158 |
|  | Actin | 2459399 | 3018226 | 1231005 | 3823932 |
| 3 | Myosin | 1459461 | 2600731 | 1215941 | 4943204 |
|  | YP170 | 2756108 | 6494741 | 3032698 | 12884352 |
|  | YP115 | 1754749 | 3609675 | 1357186 | 5286976 |
|  | YP88 | 1831570 | 3952315 | 1334686 | 7225118 |
|  | Actin | 1932864 | 2918877 | 1576602 | 4157375 |
| 4 | Myosin | 1496483 | 2831276 | 1222045 | 5728315 |
|  | YP170 | 4284651 | 10847333 | 4690193 | 20364108 |
|  | YP115 | 2081776 | 4838981 | 1710519 | 6727091 |
|  | YP88 | 2146797 | 4873520 | 1750105 | 7849487 |
|  | Actin | 1876025 | 3077507 | 1552176 | 3971231 |
| 5 | Myosin | 1750933 | 2901153 | 1302762 | 6900336 |
|  | YP170 | 7564779 | 13087132 | 5810031 | 25918524 |
|  | YP115 | 2790946 | 6068936 | 1889860 | 9015515 |
|  | YP88 | 2860264 | 6454395 | 1801919 | 11556696 |
|  | Actin | 2417315 | 3268479 | 1610410 | 4226116 |
| 6 | Myosin | 1440409 | 2662999 | 1201300 | 4946367 |
|  | YP170 | 6143591 | 14115940 | 5999738 | 23664261 |
|  | YP115 | 2803862 | 7239521 | 1941769 | 9369095 |
|  | YP88 | 2925744 | 7189501 | 1877806 | 10778685 |
|  | Actin | 2144884 | 3166945 | 1592815 | 4205424 |
| 7 | Myosin | 1965003 | 2554736 | 1249453 | 4286838 |
|  | YP170 | 10590925 | 15613456 | 6808620 | 22603066 |
|  | YP115 | 4684842 | 8999904 | 2247643 | 11161726 |
|  | YP88 | 4724766 | 8485414 | 2286227 | 10904979 |
|  | Actin | 2668496 | 2939537 | 1733254 | 3682682 |

**A.** cf. Figure 1B; Supplementary Figure 1 (continued).

| Days | Bands | N2 | | | |
| --- | --- | --- | --- | --- | --- |
|  |  | Trial 1 | Trial 2 | Trial 3 | Trial 4 |
| 8 | Myosin | 2058320 | 2792720 | 1290305 | 5001398 |
|  | YP170 | 13790909 | 17736549 | 7659179 | 23981741 |
|  | YP115 | 5762738 | 10432154 | 2448091 | 13273525 |
|  | YP88 | 5630589 | 9110250 | 2353492 | 10159696 |
|  | Actin | 2577205 | 3451229 | 1676370 | 4876394 |
| 10 | Myosin | 1968160 | 2630156 | 1232724 | 4774553 |
|  | YP170 | 14487296 | 16664875 | 8050401 | 22914559 |
|  | YP115 | 6834874 | 10650505 | 2817644 | 12043170 |
|  | YP88 | 6370116 | 9637527 | 2441657 | 12996811 |
|  | Actin | 2187257 | 2875469 | 1529294 | 4156299 |
| 12 | Myosin | 1792729 | 2627927 | 1237826 | 4179950 |
|  | YP170 | 13016375 | 17230908 | 9435196 | 22457410 |
|  | YP115 | 5532101 | 11170372 | 3052114 | 13324607 |
|  | YP88 | 5357310 | 10332324 | 2690451 | 14710700 |
|  | Actin | 1848069 | 2741872 | 1527609 | 3701567 |
| 14 | Myosin | 1565221 | 2644856 | 1253674 | 4991792 |
|  | YP170 | 10882233 | 17434428 | 10678446 | 26810631 |
|  | YP115 | 4960727 | 12138488 | 3239393 | 16995025 |
|  | YP88 | 5008176 | 10758385 | 2853698 | 15919238 |
|  | Actin | 1566109 | 2600781 | 1506739 | 4176865 |
| 16 | Myosin | 1552113 | 2531693 | 1257394 | 4686625 |
|  | YP170 | 11658104 | 17182280 | 9093160 | 24273332 |
|  | YP115 | 4932297 | 11338444 | 3287389 | 13871750 |
|  | YP88 | 5061601 | 10787912 | 2839460 | 14946286 |
|  | Actin | 1508593 | 2445632 | 1468412 | 3900133 |
| 18 | Myosin | 1868289 | 2325350 | 1256775 | 4336420 |
|  | YP170 | 15099726 | 15180150 | 7471510 | 19531194 |
|  | YP115 | 6866066 | 10669676 | 2439845 | 10628130 |
|  | YP88 | 6602961 | 9475011 | 2259027 | 11104374 |
|  | Actin | 1994222 | 2665903 | 1410963 | 3390919 |
| 20 | Myosin | 1255170 | 2033852 | 1296839 | 4214198 |
|  | YP170 | 9996476 | 13321987 | 6397060 | 17395216 |
|  | YP115 | 4337537 | 9073686 | 2511212 | 9681435 |
|  | YP88 | 4602053 | 8862193 | 2278132 | 10925941 |
|  | Actin | 1398960 | 1935940 | 1635173 | 3365375 |

**B.** cf. Figure 1D; Supplementary Figure 2.

| Days | Bands | Trial 1 | | Trial 2 | | Trial 3 | |
| --- | --- | --- | --- | --- | --- | --- | --- |
|  |  | N2 | Mated N2 | N2 | Mated N2 | N2 | Mated N2 |
| 1 | Myosin | 1075484 | 885535 | 1348432 | 1201859 | 1205202 | 1049327 |
|  | YP170 | 1191420 | 905771 | 1825193 | 1343196 | 1347610 | 1134524 |
|  | YP115 | 859952 | 675713 | 1338081 | 1185946 | 1097684 | 908814 |
|  | YP88 | 804563 | 635200 | 1366101 | 1200084 | 1175839 | 991246 |
|  | Actin | 1103622 | 700546 | 1877445 | 1646509 | 1340725 | 1258789 |
| 2 | Myosin | 999962 | 1113680 | 1512730 | 1484359 | 1092601 | 1064755 |
|  | YP170 | 1300634 | 2608586 | 3144923 | 2395028 | 1472220 | 1192487 |
|  | YP115 | 827402 | 1056526 | 1624928 | 1600639 | 905689 | 814443 |
|  | YP88 | 798839 | 983274 | 1585480 | 1566645 | 1003074 | 1039973 |
|  | Actin | 927021 | 1452416 | 2188678 | 2044092 | 1084996 | 1346972 |
| 3 | Myosin | 1322243 | 1242161 | 1730345 | 1591561 | 1225613 | 829626 |
|  | YP170 | 5220942 | 4227208 | 7069198 | 4123039 | 5563119 | 1216875 |
|  | YP115 | 1579954 | 1364201 | 2513723 | 1992599 | 2263736 | 783817 |
|  | YP88 | 1514556 | 1272023 | 2347082 | 1904765 | 2500616 | 1143127 |
|  | Actin | 1450254 | 1326207 | 2230245 | 1868408 | 1879526 | 1050082 |
| 4 | Myosin | 1552109 | 1220721 | 1921833 | 1865324 | 1150029 | 906660 |
|  | YP170 | 8089201 | 4615192 | 11681062 | 6772904 | 5569022 | 1651886 |
|  | YP115 | 2500530 | 1454111 | 3511811 | 2384160 | 2611422 | 901753 |
|  | YP88 | 2434815 | 1312146 | 3180970 | 2254232 | 4622771 | 1347083 |
|  | Actin | 1718527 | 1421900 | 2382147 | 2371310 | 1931699 | 1169934 |
| 6 | Myosin | 1480245 | 1396976 | 1606284 | 1474985 | 1239040 | 1017983 |
|  | YP170 | 9929499 | 3842622 | 8685146 | 2991347 | 8738847 | 1595176 |
|  | YP115 | 2520684 | 1535520 | 2945648 | 1696696 | 4421594 | 1035099 |
|  | YP88 | 2456071 | 1437924 | 2673947 | 1732038 | 4519202 | 1477861 |
|  | Actin | 1540118 | 1321529 | 2172687 | 2268617 | 1646311 | 1423123 |
| 8 | Myosin | 1482585 | 1242355 | 1546047 | 1327611 | 1160381 | 1215798 |
|  | YP170 | 10718993 | 3487516 | 10113593 | 2619009 | 7774495 | 1583508 |
|  | YP115 | 3228429 | 1344284 | 3125743 | 1464696 | 3466404 | 1220635 |
|  | YP88 | 2631420 | 1282503 | 3058690 | 1559514 | 4173531 | 1641802 |
|  | Actin | 1665001 | 1531538 | 2206171 | 2052579 | 1443029 | 1462714 |

**C.** cf. Figure 1E; Supplementary Figure 3.

| Days | Bands | Trial 1 | | Trial 2 | | Trial 3 | |
| --- | --- | --- | --- | --- | --- | --- | --- |
|  |  | N2 | *fog-2* | N2 | *fog-2* | N2 | *fog-2* |
| 1 | Myosin | 1702323 | 2520792 | 2335265 | 3088163 | 5108618 | 6116321 |
|  | YP170 | 2026890 | 5034641 | 1768932 | 6840740 | 4336182 | 11426954 |
|  | YP115 | 1770923 | 3168794 | 1840471 | 3767088 | 3018114 | 7390500 |
|  | YP88 | 1810026 | 3195058 | 1878152 | 4092688 | 3307470 | 7776001 |
|  | Actin | 2390422 | 3725608 | 3279344 | 3529724 | 7193830 | 8382970 |
| 2 | Myosin | 1828405 | 2449728 | 3271147 | 2955401 | 7949542 | 7451614 |
|  | YP170 | 3363331 | 11536586 | 3627675 | 9337159 | 8575919 | 19307165 |
|  | YP115 | 1931312 | 4515193 | 2111390 | 4752153 | 6105685 | 9649840 |
|  | YP88 | 1947222 | 4146324 | 2409556 | 4972518 | 7910818 | 10016733 |
|  | Actin | 2446888 | 4260261 | 3435901 | 3921332 | 9382312 | 9471206 |
| 3 | Myosin | 2030541 | 3124106 | 2748789 | 2866002 | 7301121 | 7008667 |
|  | YP170 | 6990169 | 17178007 | 6452960 | 10800988 | 17944076 | 20480773 |
|  | YP115 | 2777904 | 6560642 | 3477228 | 5342587 | 8206087 | 10048940 |
|  | YP88 | 2676144 | 5790250 | 3949964 | 5768317 | 8755407 | 10492773 |
|  | Actin | 2776625 | 4439908 | 3450171 | 4517020 | 8735754 | 9560178 |
| 4 | Myosin | 2458658 | 2667862 | 2333184 | 2185952 | 6165631 | 6030199 |
|  | YP170 | 12372710 | 16265126 | 6934562 | 10390048 | 22173939 | 21365284 |
|  | YP115 | 4055560 | 6179397 | 3760606 | 5330177 | 7948175 | 11360326 |
|  | YP88 | 3639326 | 4788397 | 4273644 | 5710591 | 8728258 | 11908215 |
|  | Actin | 3091634 | 4057242 | 3545261 | 4137410 | 8322548 | 9305224 |
| 5 | Myosin | 2338074 | 3006232 | 2403089 | 2345121 | 5832681 | 5356808 |
|  | YP170 | 12746994 | 19172576 | 7400901 | 10802858 | 23117523 | 22775240 |
|  | YP115 | 4210332 | 7630276 | 3933522 | 5227510 | 9308264 | 10091540 |
|  | YP88 | 3610657 | 5950230 | 4522831 | 5540627 | 9092821 | 11161150 |
|  | Actin | 2758990 | 4515952 | 3447166 | 3538492 | 8464058 | 8229262 |
| 6 | Myosin | 2096502 | 3313550 | 2317259 | 2306881 | 5352548 | 5201734 |
|  | YP170 | 12884259 | 22225328 | 8150241 | 10137494 | 23658897 | 24359349 |
|  | YP115 | 4107968 | 9921344 | 4272970 | 6071237 | 8916147 | 12022305 |
|  | YP88 | 3847621 | 9030441 | 4904191 | 6725699 | 10240017 | 11377534 |
|  | Actin | 2890742 | 4898833 | 3982278 | 3985060 | 8619614 | 8607054 |

**D.** cf. Supplementary Figure 4.

| Days | Bands | Trial 1 | | Trial 2 | | Trial 3 | |
| --- | --- | --- | --- | --- | --- | --- | --- |
|  |  | L4440 | *rme-2*  RNAi | L4440 | *rme-2*  RNAi | L4440 | *rme-2*  RNAi |
| 1 | Myosin | 20747554 | 20763033 | 4098752 | 4011209 | 3559027 | 3580464 |
|  | YP170 | 21037962 | 21475891 | 4485077 | 5220160 | 3882251 | 4607815 |
|  | YP115 | 20865890 | 20927912 | 4248130 | 4488068 | 3579615 | 3819471 |
|  | YP88 | 20943240 | 20933583 | 4532181 | 4714703 | 3813932 | 3988116 |
|  | Actin | 21109429 | 20991148 | 5152650 | 5042937 | 4386238 | 4292881 |
| 4 | Myosin | 21764216 | 22380690 | 4023994 | 4089513 | 4624895 | 5051990 |
|  | YP170 | 61866906 | 91474390 | 11497677 | 19170016 | 12652906 | 19506819 |
|  | YP115 | 31356138 | 47771483 | 7124171 | 9249128 | 6131267 | 10759501 |
|  | YP88 | 31341665 | 44601162 | 7111584 | 9002268 | 6121565 | 10081239 |
|  | Actin | 22338417 | 23033366 | 5167683 | 5132231 | 5495569 | 5959018 |
| 6 | Myosin | 22341813 | 22400890 | 4092225 | 4075445 | 5381913 | 5379685 |
|  | YP170 | 88891199 | 100704297 | 16975269 | 19455452 | 21831329 | 26712687 |
|  | YP115 | 44483854 | 51073501 | 8072569 | 10009723 | 9561924 | 11232066 |
|  | YP88 | 39736537 | 53517818 | 8171521 | 10033444 | 9168876 | 12810907 |
|  | Actin | 22790267 | 22653914 | 5196994 | 5113155 | 5959323 | 5723117 |
| 8 | Myosin | 22272273 | 22060367 | 4029784 | 4004918 | 5134690 | 4768352 |
|  | YP170 | 99916652 | 115997475 | 17069507 | 18804541 | 22830837 | 26182778 |
|  | YP115 | 54104317 | 54548024 | 8008952 | 12061741 | 9575341 | 9998618 |
|  | YP88 | 53038490 | 53312854 | 8094054 | 10028991 | 9595638 | 11187615 |
|  | Actin | 22567497 | 22303182 | 5196710 | 5134248 | 5607842 | 5322030 |

**E.** cf. Supplementary Figure 5.

| Days | Bands | Trial 1 | | Trial 2 | | Trial 3 | |
| --- | --- | --- | --- | --- | --- | --- | --- |
|  |  | *fog-2* | *fog-2* x  *rrf-3* | *fog-2* | *fog-2* x  *rrf-3* | *fog-2* | *fog-2* x  *rrf-3* |
| 1 | Myosin | 1509984 | 1637886 | 1524628 | 1637886 | 2098393 | 2260838 |
|  | YP170 | 4610674 | 4717325 | 3613940 | 4736305 | 4570427 | 4897872 |
|  | YP115 | 2773162 | 3083159 | 2766704 | 3083159 | 3730777 | 4180903 |
|  | YP88 | 3190554 | 3133767 | 3118131 | 3247084 | 4284211 | 4451405 |
|  | Actin | 2275043 | 2454653 | 2275043 | 2454653 | 3251721 | 3512886 |
| 4 | Myosin | 1403046 | 1135867 | 1383696 | 1129161 | 1907731 | 1536345 |
|  | YP170 | 9011399 | 6256612 | 8029096 | 6260832 | 8675806 | 8479490 |
|  | YP115 | 3211379 | 2820110 | 3546340 | 2820110 | 4522581 | 3702187 |
|  | YP88 | 3347761 | 3165783 | 3317435 | 3128524 | 4446813 | 4184896 |
|  | Actin | 2282903 | 2247300 | 2274857 | 2248815 | 3236933 | 3195853 |
| 6 | Myosin | 1830865 | 1793597 | 2030865 | 1832315 | 3623189 | 2490180 |
|  | YP170 | 14083636 | 11985638 | 13559342 | 12347571 | 17530882 | 16340368 |
|  | YP115 | 6998333 | 5529831 | 5579812 | 4774569 | 7229729 | 6680973 |
|  | YP88 | 5510266 | 5153409 | 5542603 | 5201011 | 7234197 | 6709199 |
|  | Actin | 3376030 | 3192560 | 3371542 | 3199540 | 4725040 | 4468897 |
| 8 | Myosin | 1941627 | 1720693 | 1941494 | 3059897 | 2631469 | 4225993 |
|  | YP170 | 14249184 | 14238603 | 13608236 | 13676054 | 16437952 | 30509107 |
|  | YP115 | 6796845 | 7485565 | 5662551 | 5508325 | 6094596 | 7321105 |
|  | YP88 | 5774032 | 7688890 | 4778215 | 5650579 | 6274912 | 7525721 |
|  | Actin | 3296832 | 3796398 | 3296832 | 3825056 | 4609246 | 5376771 |

**F.** cf. Supplementary Figure 6.

| Days | Bands | Trial 1 | | Trial 2 | | Trial 3 | |
| --- | --- | --- | --- | --- | --- | --- | --- |
|  |  | *fog-2* | Mated  *fog-2* | *fog-2* | Mated  *fog-2* | *fog-2* | Mated  *fog-2* |
| 1 | Myosin | 2274603 | 1649263 | 2766981 | 1862269 | 2835045 | 2765804 |
|  | YP170 | 4173554 | 2584810 | 5895727 | 3932244 | 5973710 | 4598506 |
|  | YP115 | 2494372 | 1801839 | 3843217 | 2698432 | 3900612 | 3458395 |
|  | YP88 | 2417625 | 1887091 | 3972491 | 2967572 | 4025988 | 3673696 |
|  | Actin | 2697479 | 2409467 | 4753737 | 4054470 | 4792565 | 4564563 |
| 2 | Myosin | 2178058 | 1550226 | 2721398 | 2943462 | 3443915 | 3526304 |
|  | YP170 | 11325006 | 2557340 | 11748167 | 5304775 | 12981034 | 6572860 |
|  | YP115 | 4020549 | 1311563 | 5009837 | 3482294 | 5612948 | 3969721 |
|  | YP88 | 3433862 | 1421193 | 4858787 | 3682046 | 5418779 | 4136167 |
|  | Actin | 3529095 | 2521913 | 4991427 | 5133012 | 5396634 | 5462914 |
| 3 | Myosin | 2600788 | 2204018 | 3647424 | 3268729 | 4025585 | 3947251 |
|  | YP170 | 15160986 | 4974240 | 19195027 | 7396762 | 20047350 | 8213783 |
|  | YP115 | 5390483 | 3062700 | 7730801 | 4268966 | 8106659 | 4783158 |
|  | YP88 | 4711321 | 3032391 | 6869180 | 4161076 | 7190011 | 4641535 |
|  | Actin | 3771514 | 3768220 | 5108301 | 4687329 | 5320555 | 5034782 |
| 4 | Myosin | 2216800 | 2536631 | 3118924 | 3598453 | 3925914 | 3767607 |
|  | YP170 | 13505117 | 9229454 | 19025134 | 11363599 | 20992480 | 9618659 |
|  | YP115 | 5045960 | 3952011 | 7312834 | 5422371 | 8054536 | 5564144 |
|  | YP88 | 3885586 | 3612778 | 5691207 | 5345015 | 6257295 | 5477047 |
|  | Actin | 3180909 | 3866473 | 4933576 | 5088945 | 5394623 | 5185698 |
| 6 | Myosin | 2515584 | 1351654 | 3496880 | 3032917 | 3721954 | 3817613 |
|  | YP170 | 17012423 | 3661638 | 20332729 | 7535851 | 20883364 | 7558546 |
|  | YP115 | 6197607 | 1574215 | 9062944 | 4408030 | 9326632 | 5131205 |
|  | YP88 | 4625935 | 1727989 | 7274525 | 4174120 | 7468050 | 4788728 |
|  | Actin | 3701347 | 2461598 | 5330558 | 5145925 | 5459446 | 5594064 |
| 8 | Myosin | 2602867 | 1287170 | 4024232 | 3446822 | 4329157 | 3399860 |
|  | YP170 | 19021818 | 5014641 | 22428838 | 9310759 | 23160641 | 8294231 |
|  | YP115 | 8861412 | 2843844 | 10981275 | 5499647 | 11351444 | 5831883 |
|  | YP88 | 8144510 | 2884514 | 9916372 | 6158234 | 10240904 | 6524462 |
|  | Actin | 4013031 | 2179899 | 5784636 | 4775093 | 5943733 | 4981848 |

**G.** cf. Figure 2B.

| Days | Bands | Trial 1 | | | | Trial 2 | | | | Trial 3 | | | |
| --- | --- | --- | --- | --- | --- | --- | --- | --- | --- | --- | --- | --- | --- |
|  |  | L4440 | *vit-5* | *vit-6* | *vit-5,-6* | L4440 | *vit-5* | *vit-6* | *vit-5,-6* | L4440 | *vit-5* | *vit-6* | *vit-5,-6* |
|  |  |  | RNAi | RNAi | RNAi |  | RNAi | RNAi | RNAi |  | RNAi | RNAi | RNAi |
| 1 | Myosin | 3496110 | 2346720 | 3698474 | 3624599 | 3775367 | 3895119 | 3727864 | 4209094 | 2525569 | 2598983 | 2394949 | 2273078 |
|  | YP170 | 3823496 | 1828672 | 3619792 | 3223911 | 5950894 | 3742942 | 6677036 | 4376205 | 3192637 | 2428979 | 3154489 | 2125802 |
|  | YP115 | 2896551 | 1974512 | 2969783 | 2930720 | 5079114 | 5325368 | 4690429 | 4417085 | 3631664 | 3689368 | 2876697 | 2997741 |
|  | YP88 | 3359603 | 2629003 | 3383912 | 3250625 | 5077109 | 5204823 | 4751980 | 4672397 | 3778680 | 4199295 | 3367586 | 3156993 |
|  | Actin | 3582559 | 2714150 | 3602378 | 3242166 | 4694941 | 4660024 | 4551081 | 4669298 | 4508622 | 4708535 | 4142106 | 3882321 |
| 4 | Myosin | 4609349 | 4613611 | 3959245 | 4382265 | 4187812 | 3809159 | 3960584 | 3520477 | 2970743 | 2784689 | 2477350 | 2222636 |
|  | YP170 | 10237082 | 4647043 | 17297684 | 4862666 | 17460417 | 3681914 | 17041257 | 3258876 | 11238797 | 2592013 | 13089736 | 2116633 |
|  | YP115 | 6807042 | 10420566 | 3074970 | 3352076 | 12029506 | 13272205 | 4526634 | 3918057 | 7440233 | 10729237 | 2610666 | 2939558 |
|  | YP88 | 8162868 | 10918118 | 4280455 | 4529129 | 11851858 | 15680613 | 4906509 | 4641462 | 7232126 | 9407567 | 3201400 | 3358193 |
|  | Actin | 5070057 | 4433480 | 4089907 | 4187346 | 5136528 | 5073410 | 4989819 | 5153514 | 4329911 | 4453343 | 4437610 | 4587701 |
| 7 | Myosin | 4179606 | 3922856 | 2987839 | 4425476 | 3308225 | 3916096 | 4018690 | 4099184 | 2884437 | 2645553 | 2515499 | 2438021 |
|  | YP170 | 15088776 | 3763603 | 13104341 | 4771984 | 20646090 | 3855732 | 28834442 | 4029174 | 15176997 | 2580387 | 17259614 | 2326438 |
|  | YP115 | 6742675 | 9678924 | 2474949 | 3568732 | 12511436 | 14155147 | 4353834 | 4345004 | 9801530 | 15600126 | 3222729 | 3129439 |
|  | YP88 | 6792385 | 10357383 | 3848104 | 4387316 | 14543489 | 15491925 | 4718150 | 4694240 | 9311876 | 15828994 | 3638986 | 3428375 |
|  | Actin | 3940680 | 3578204 | 3162223 | 4543026 | 4874811 | 5337817 | 5343710 | 5707731 | 4702749 | 4850907 | 4645217 | 4641184 |
| 11 | Myosin | 4540797 | 3406663 | 4194501 | 4501171 | 4092147 | 3441502 | 4023439 | 4007752 | 2517934 | 2452622 | 2783814 | 2594768 |
|  | YP170 | 17852630 | 3481877 | 19062087 | 4862092 | 24241026 | 3227508 | 29854020 | 3583720 | 12334960 | 2544663 | 20425007 | 2338517 |
|  | YP115 | 7596271 | 7681841 | 3189204 | 3570482 | 15350627 | 18452038 | 4225403 | 4306835 | 10629686 | 12692113 | 3340517 | 3156123 |
|  | YP88 | 9880138 | 9005964 | 4207638 | 4489958 | 13908168 | 17694110 | 4672217 | 4601371 | 8982051 | 13878720 | 3364427 | 3303572 |
|  | Actin | 4346675 | 3719944 | 4159956 | 4685410 | 5511593 | 4994943 | 5364946 | 5304130 | 4273243 | 4434287 | 4611390 | 4745721 |

These tables show densitometric measurements of bands on Coomassie Blue stained protein gels.
