# Supplementary material for: Production of YP170 Vitellogenins Promotes Intestinal Senescence in Caenorhabditis elegans
Source: J Gerontol A Biol Sci Med Sci. 2019 Mar 15;74(8):1180–8. doi: 10.1093/gerona/glz067 (PMC6625598; doi:10.1093/gerona/glz067)
Supplement: glz067_suppl_Supplementary_Material [file glz067_suppl_supplementary_material.docx]

**Supplementary Material**

**Production of YP170 vitellogenins promotes intestinal senescence in *C. elegans***

**Thanet Sornda, Marina Ezcurra, Carina Kern, Evgeniy R. Galimov, Catherine Au, Yila de la Guardia and David Gems**

**Supplementary Figure 1**. Age accumulation of YP in N2, individual trials.

**Supplementary Figure 2**. Age accumulation of YP in N2 mated with *fog-2* males.

**Supplementary Figure 3**. Age accumulation of YP in N2 hermaphrodites and *fog-2* females.

**Supplementary Figure 4**. Age accumulation of YP in N2 with *rme-2* RNAi.

**Supplementary Figure 5.** Age accumulation of YP in *fog-2* females mated with sperm-defective *rrf-3* males.

**Supplementary Figure 6.** Age accumulation of YP in *fog-2* females mated with *fog-2* males.

**Supplementary Figure 7.** Effects of *vit-6* RNAi on survival, individual trials.

**Supplementary Figure 8**. Effects of *vit* RNAi with 40 mM paraquat and with 7.5 mM *t*-BOOH on survival (combined trials).

**Supplementary Table 1**: Summary statistics for lifespan analyses.

**Supplementary Table 2**. Summary statistics for Oxr tests (paraquat).

**Supplementary Table 3**. Summary statistics for Oxr tests (*t*-BOOH).

**Supplementary Table 4**. Relative abundance of *vit* mRNAs.

**Supplementary Table 5**. Relative representation of *vit* mRNAs on ribosomes.

**Supplementary Dataset 1**. Raw YP measurement data.

**Supplementary Dataset 2**. Ziehm table with full mortality data.

**
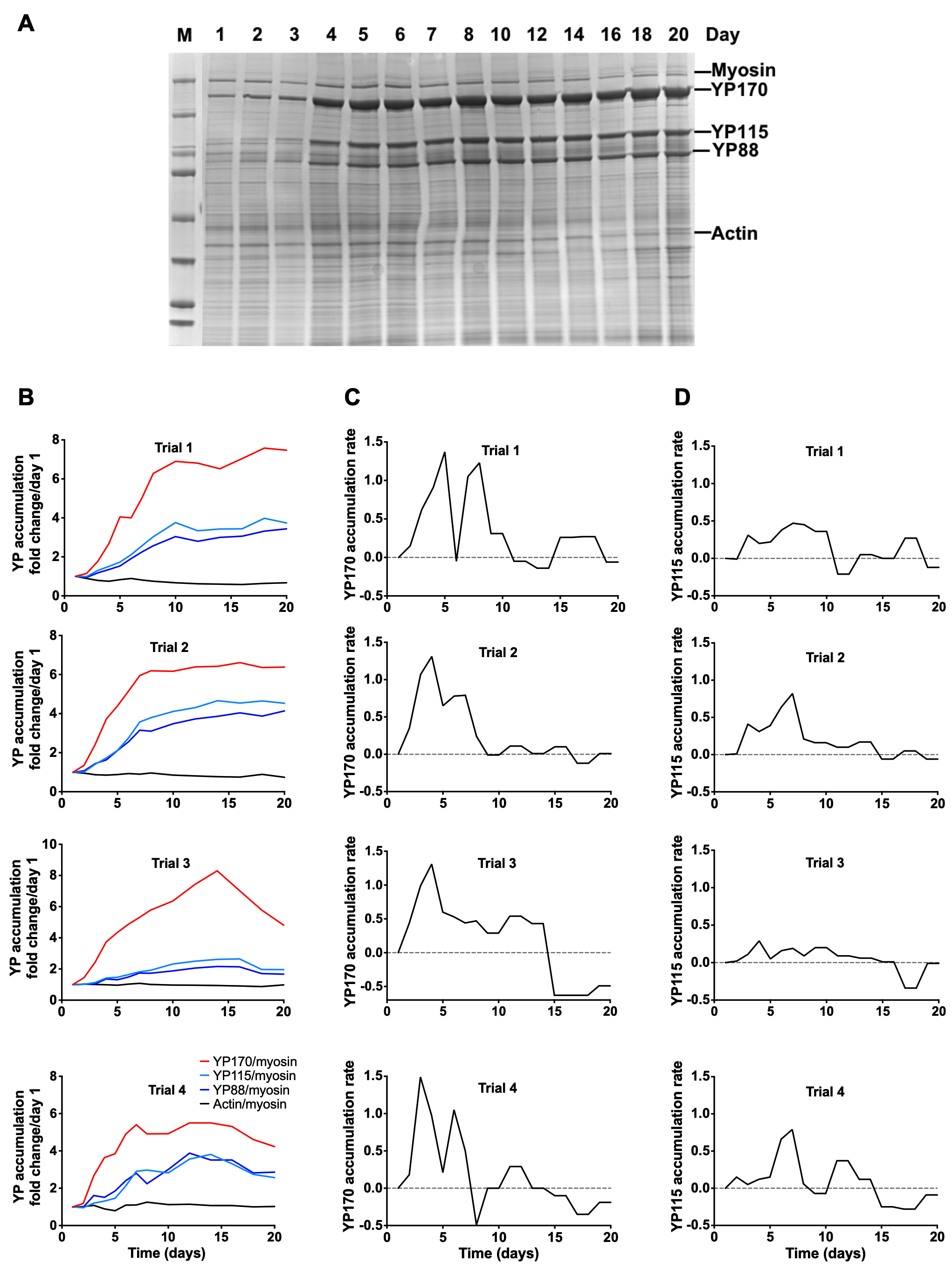
**

**Supplementary Figure 1**. Dynamics of age accumulation of YP in N2 hermaphrodites: individual trials. (**A**) Protein gel showing YP accumulation in N2 hermaphrodites. (**B**) Quantification of N2 hermaphrodite yolk (YP170, YP115, YP88) accumulation, data from 4 individual trials. (**C, D**) N2 daily increase in YP levels (accumulation rate), data from 4 individual trials. (**C**) YP170. (**D**) YP115. (**B**-**D**) Data not adjusted for age changes in intestinal size.

**
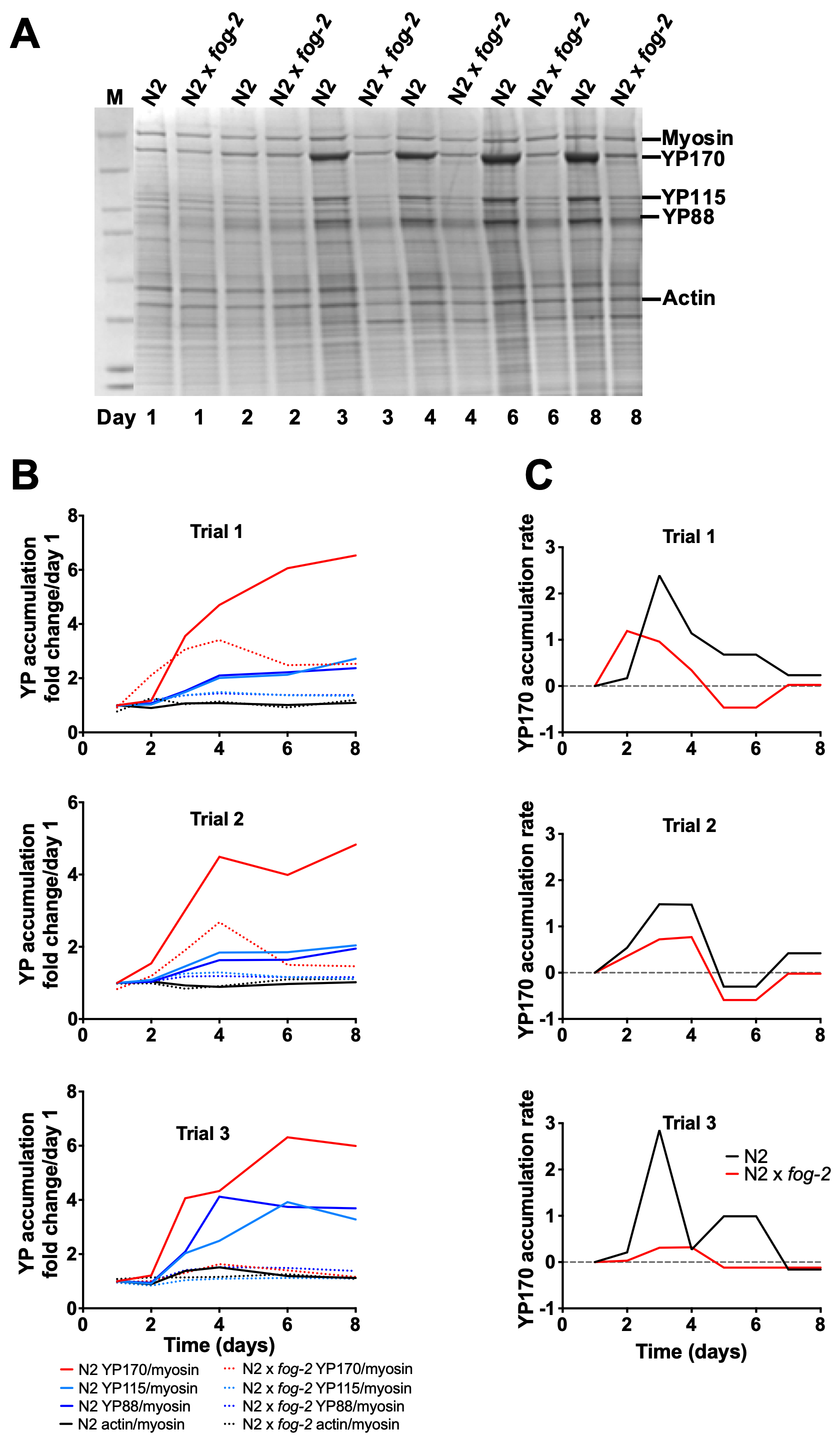
**

**Supplementary Figure 2**. Dynamics of age accumulation of YP in N2 mated with *fog-2* males. (**A**) Representative protein gel showing YP accumulation in unmated N2 hermaphrodites and N2 hermaphrodites mated with *fog-2* males (**B**) Quantification of yolk (YP170, YP115, YP88) accumulation, data from 3 individual trials. (**C**) YP170 accumulation rate, data from 3 individual trials. (**B**, **C**) Data not adjusted for age changes in intestinal size.

**
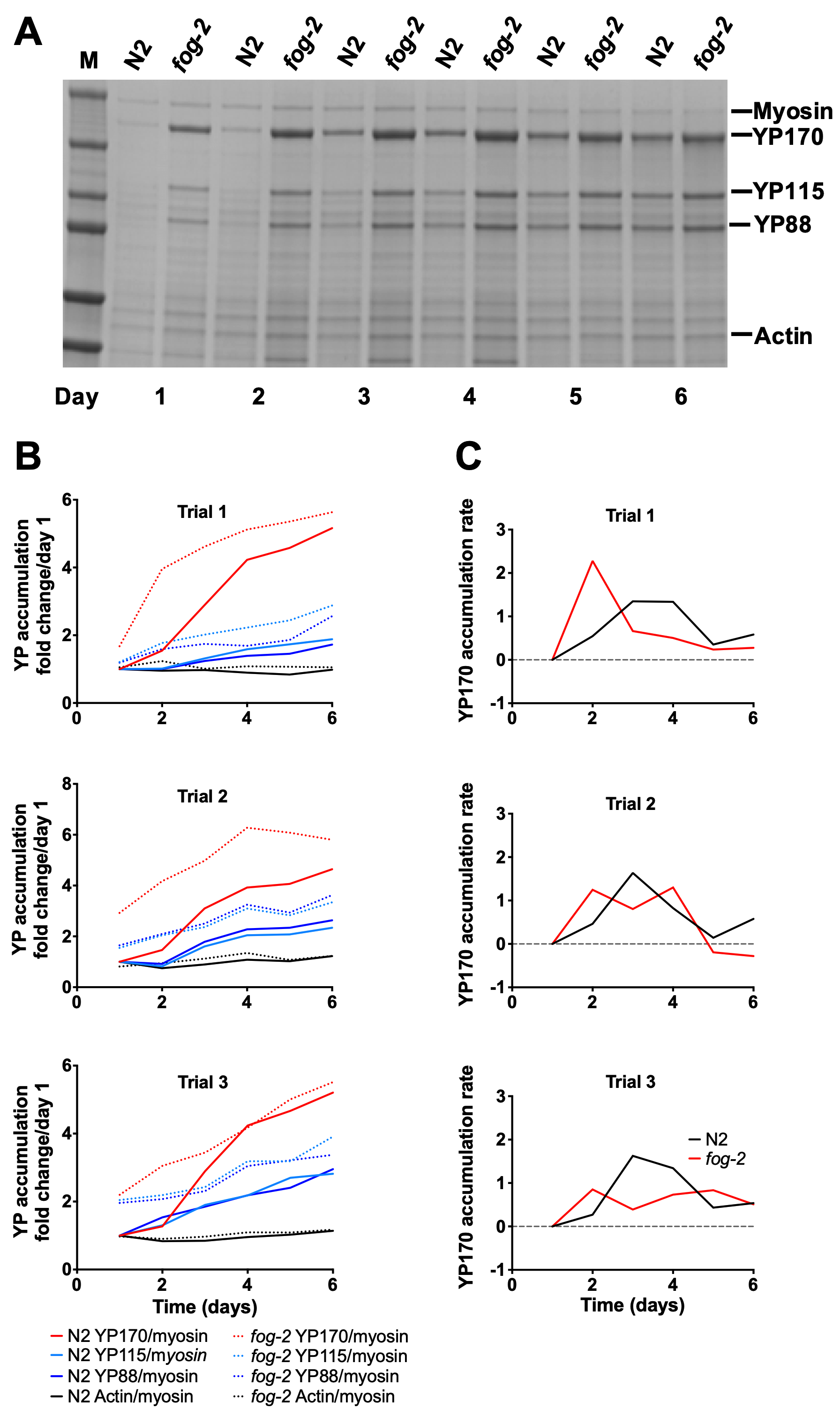
**

**Supplementary Figure 3**. Dynamics of age accumulation of YP between N2 hermaphrodites and *fog-2* females. (**A**) Representative protein gel showing YP accumulation in N2 hermaphrodites and *fog-2* females. (**B**) Quantification of yolk (YP170, YP115, YP88) accumulation, data from 3 individual trials. (**C**) YP170 accumulation rate, data from 3 individual trials. (**B**, **C**) Data not adjusted for age changes in intestinal size.


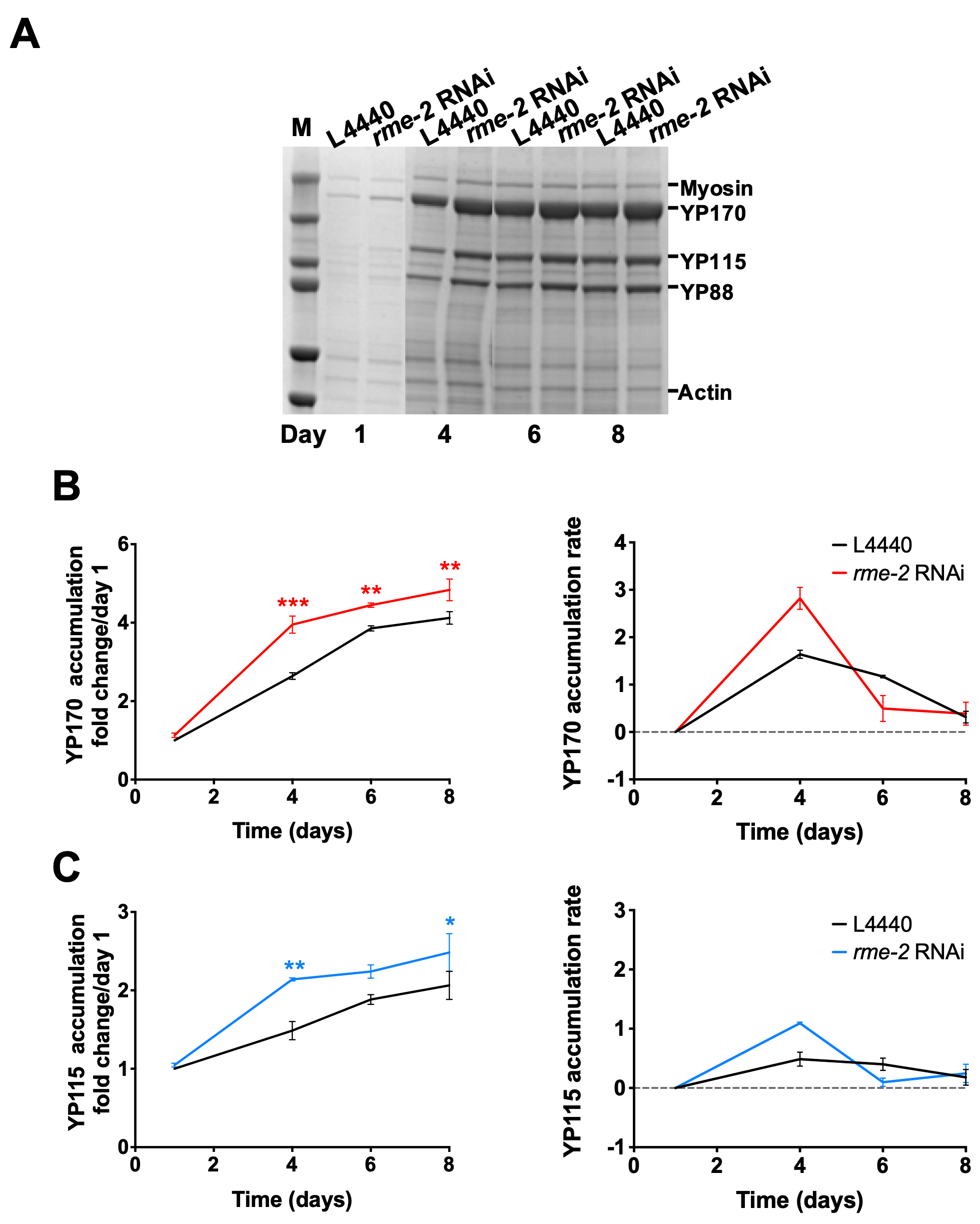


**Supplementary Figure 4**. Dynamics of age accumulation of YP in N2 following *rme-2* RNAi. (**A**) Representative protein gel showing YP accumulation in N2 following *rme-2* RNAi and in control L4440 RNAi. (**B, C**) Quantification of YP accumulation (left) and YP accumulation rate (right). (**B**) YP170. (**C**) YP115. Data are mean ± SEM, age-matched comparison, * *p* < 0.05, ** *p* < 0.01, *** *p* < 0.001. (**B**, **C**) Data not adjusted for age changes in intestinal size.

**
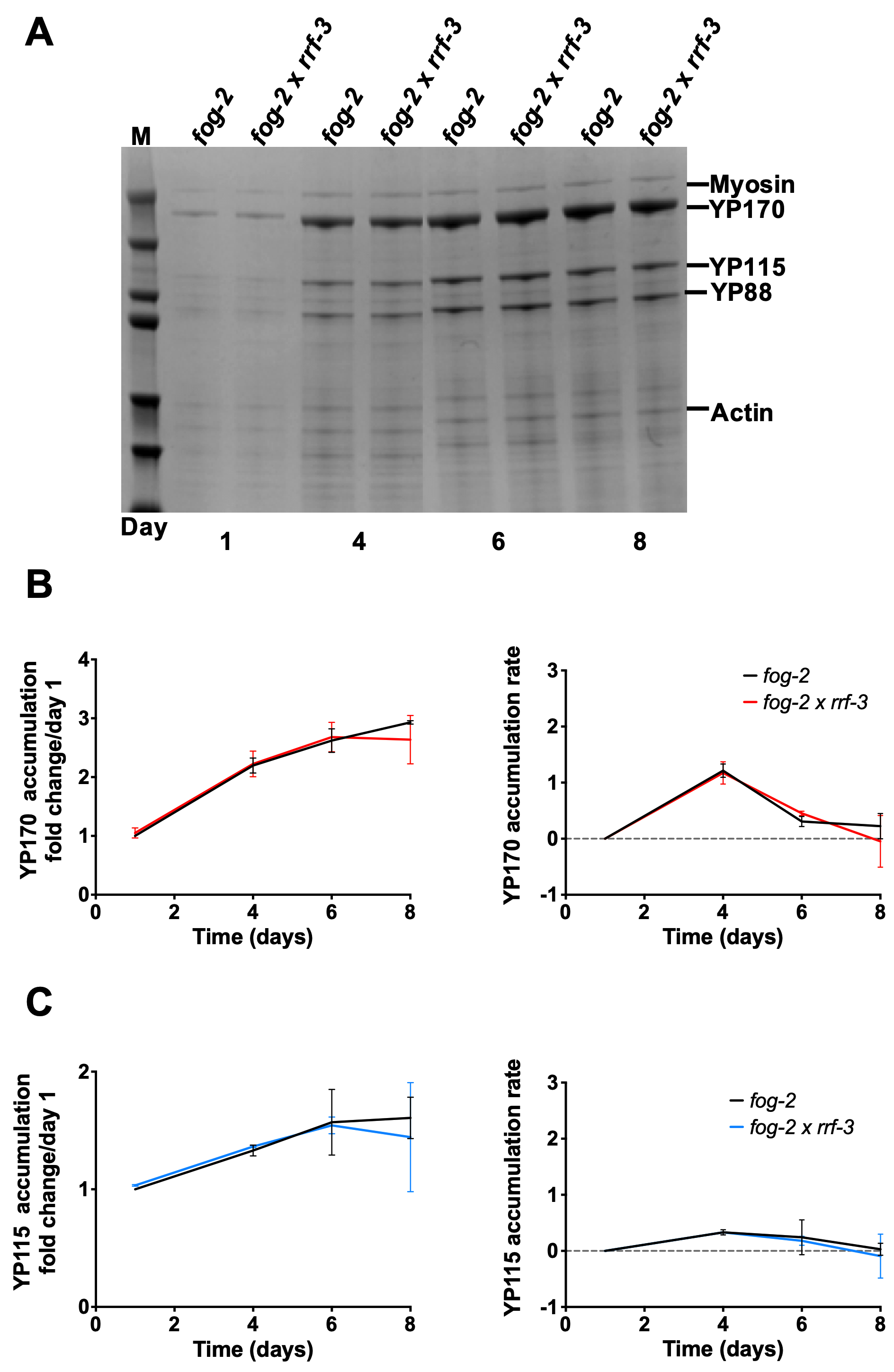
**

**Supplementary Figure 5.** Dynamics of age accumulation of YP in *fog-2* females mated with sperm defective *rrf-3* males. (**A**) Representative protein gel showing YP accumulation in unmated *fog-2* females and *fog-2* females mated with sperm defective *rrf-3* males. (**B, C**) Quantification of YP accumulation (left) and YP accumulation rate (right). (**B**) YP170. (**C**) YP115. Data are mean ± SEM, age-matched comparison. (**B**, **C**) Data not adjusted for age changes in intestinal size.

**
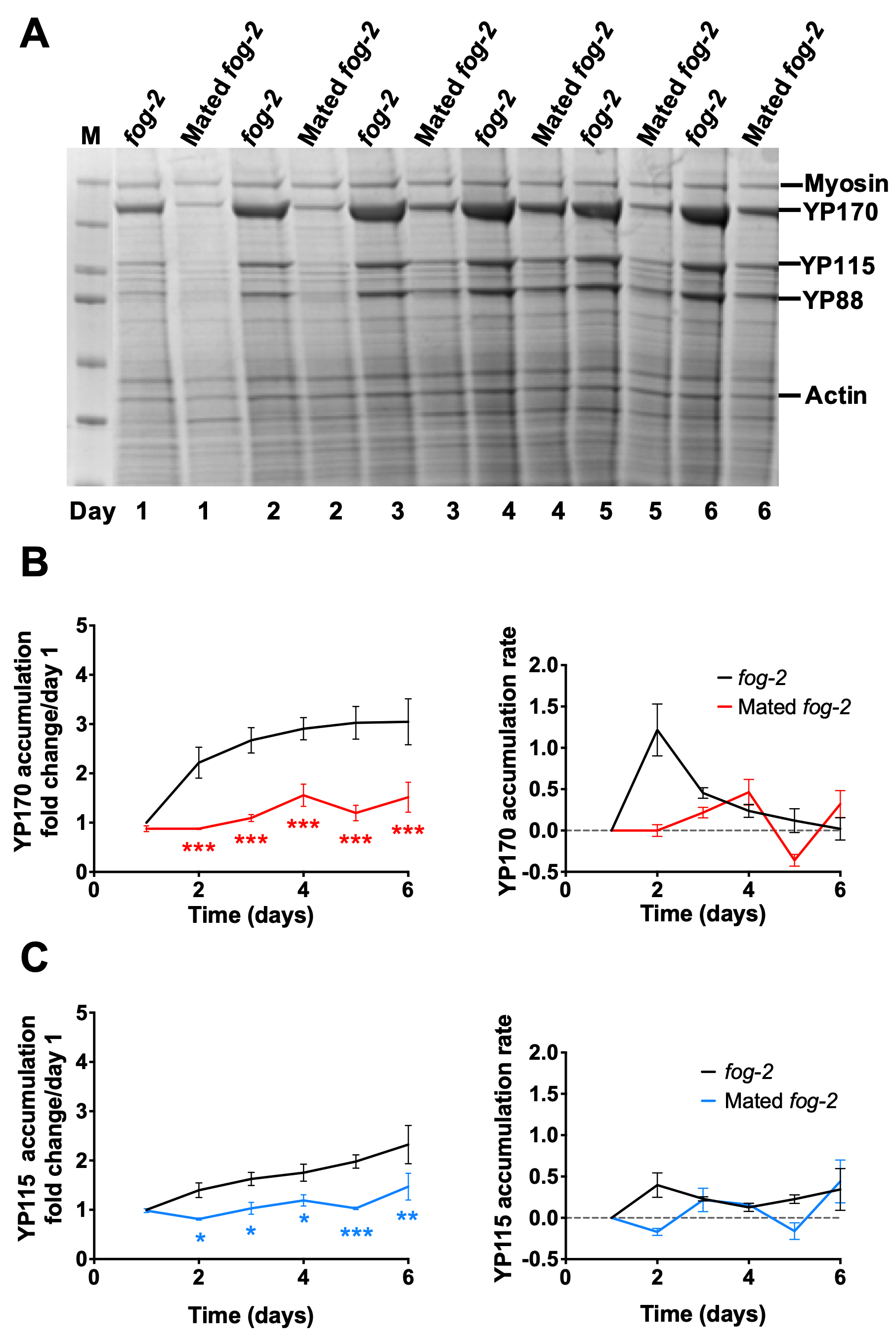
**

**Supplementary Figure 6.** Dynamics of age accumulation of YP in *fog-2* females mated with *fog-2* males. (**A**) Representative protein gel showing YP accumulation in unmated *fog-2* females and *fog-2* females mated with *fog-2* males. (**B, C**) Quantification of YP accumulation (left) and YP accumulation rate (right). (**B**) YP170. (**C**) YP115. Data are mean ± SEM, age-matched comparison, * *p* < 0.05, ** *p* < 0.01, *** *p* < 0.001. (**B**, **C**) Data not adjusted for age changes in intestinal size.

**
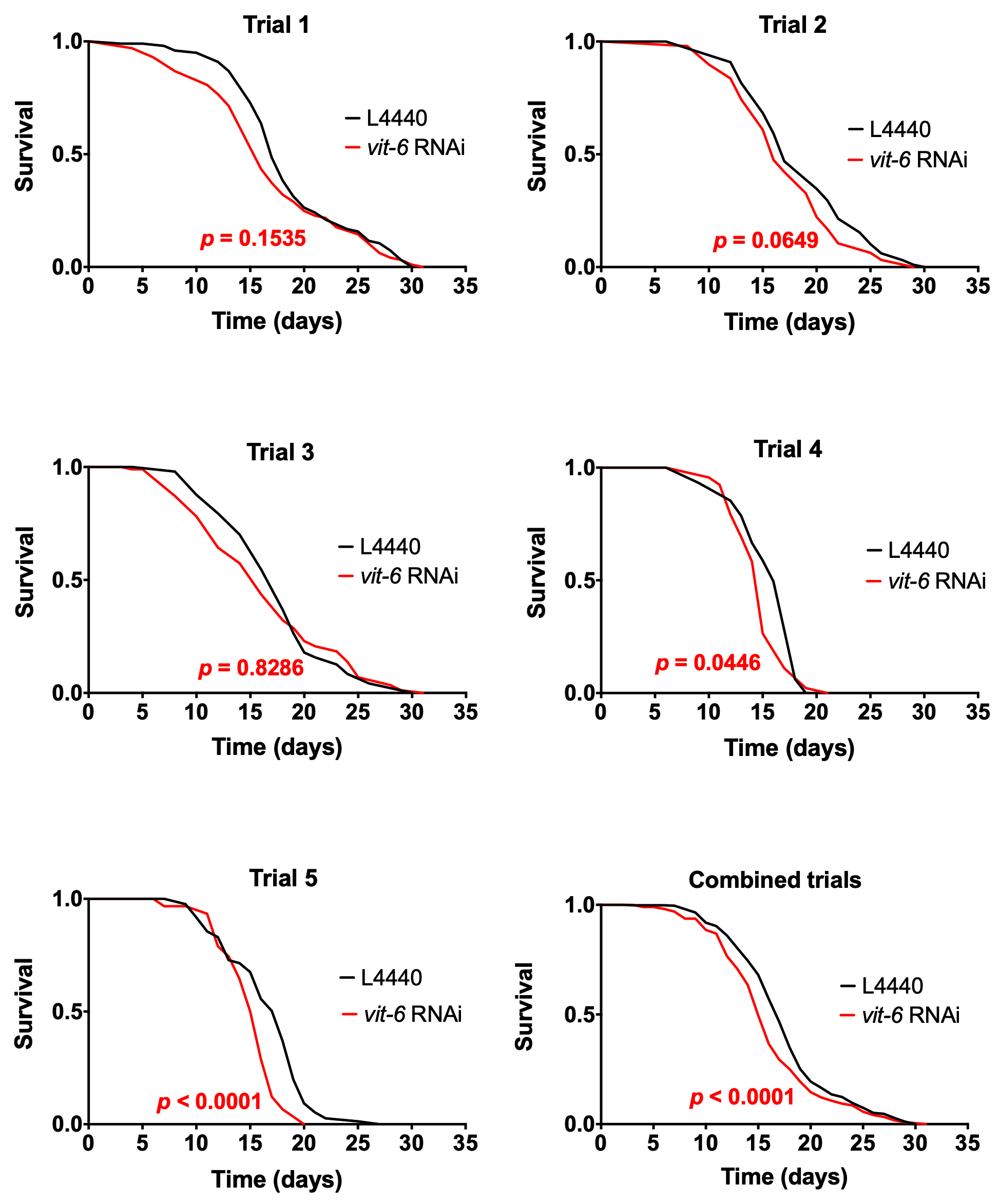
**

**Supplementary Figure 7**. Effects of *vit-6* RNAi on lifespan. For statistics, see Supplementary Table 1.

**
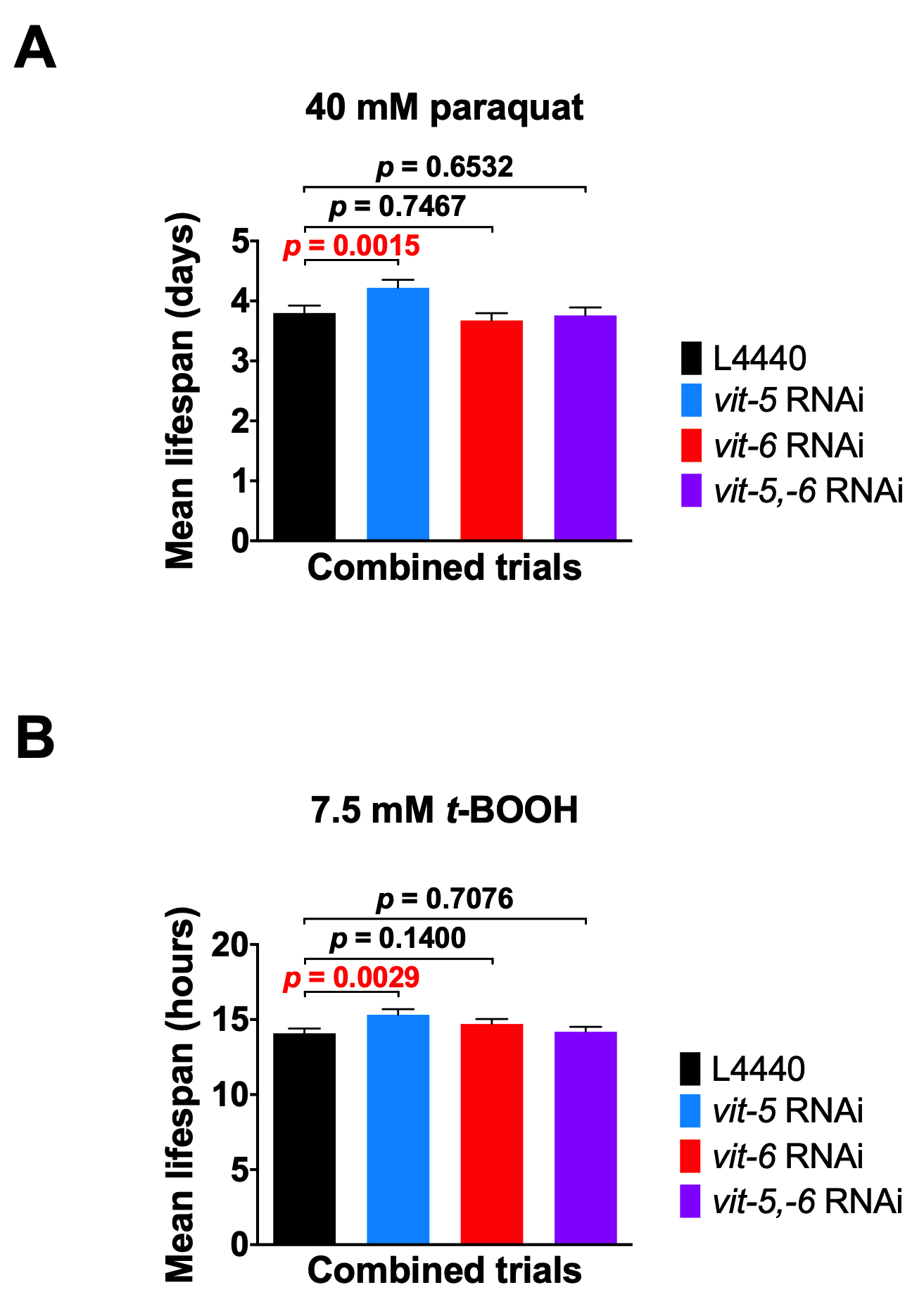
**

**Supplementary Figure 8**. Effects of *vit* RNAi with 40 mM paraquat (**A**) and 7.5 mM *t*-BOOH (**B**) on survival (combined trials). Data are mean ± SEM from 6 individual trials (**A**) and 3 individual trials (**B**). Log-rank test, compared to control. For statistics, see Supplementary Table 2 (**A**) and 3 (**B**).

**Supplementary Table 1**: Summary statistics for lifespan analyses (cf. Figure 4B)

| **Strain/**  **conditions** | **Number**  **of deaths/**  **censored^1^** | **Mean**  **[median]**  **lifespan (days)** | **% change vs.**  **control** | ***p* vs.**  **control**  **(log rank)** | **% change**  **vs.**  ***vit-5* RNAi** | ***p* vs.**  ***vit-5* RNAi**  **(log rank)** |
| --- | --- | --- | --- | --- | --- | --- |
| N2  L4440 (RNAi control) | [C] 436/58  [1] 98/2  [2] 98/2  [3] 96/4  [4] 67/27  [5] 77/23 | 17.4 [17]  18.4 [17]  18.4 [17]  17.3 [18]  15.5 [16]  16.6 [16] |  |  |  |  |
| N2  *vit-5* RNAi | [C] 286/14  [1] 98/2  [2] 97/3  [3] 91/9 | 19.2 [19]  19.2 [19]  19.6 [20]  18.8 [19] | +10.4 [+12]  +4.4 [+12]  +6.3 [+18]  +8.7 [+6] | **<0.0001**  0.1764  0.0903  **0.0414** |  |  |
| N2  *vit-6* RNAi | [C] 462/39  [1] 97/3  [2] 96/4  [3] 88/12  [4] 91/10  [5] 90/10 | 16.0 [15]  16.6 [16]  17.1 [16]  16.4 [16]  14.7 [15]  15.0 [15] | -8.2 [-12]  -9.7 [-6]  -7.1 [-6]  -5.2 [-11]  -5.3 [-6]  -9.5 [-6] | **<0.0001**  0.1535  0.0649  0.8286  **0.0446**  **<0.0001** | -16.9 [-21]  -13.5 [-16]  -12.6 [-20]  -12.8 [-16] | **<0.0001**  **0.0110**  **0.0006**  0.0662 |
| N2  *vit-5,-6* RNAi | [C] 295/5  [1] 99/1  [2] 98/2  [3] 98/2 | 20.4 [20]  22.0 [22]  19.7 [20]  19.7 [20] | +17.4 [+18]  +19.4 [+29]  +6.6 [+18]  +13.6 [+11] | **<0.0001**  **<0.0001**  0.0943  **0.0014** | +6.3 [+5]  +14.3 [+16]  +0.2 [0]  +4.5 [+5] | **0.0031**  **0.0006**  0.959  0.2195 |

^1^Numbers in parenthesis indicate individual trials. [C], combined (pooled) data from all trials.

| **Strain/**  **conditions** | **Number of deaths/censored^1^** | **Mean [median] survival (hr)** | **% change vs.**  **control** | ***p* vs. control**  **(log rank)** |
| --- | --- | --- | --- | --- |
| N2  L4440 (RNAi control) | [C] 139/11  [1] 23/2  [2] 24/1  [3] 20/5  [4] 22/3  [5] 25/0  [6] 25/0 | 3.8 [4]  3.7 [4]  3.9 [3]  4.1 [5]  4.0 [4]  3.4 [3]  3.7 [4] |  |  |
| N2  *vit-5* RNAi | [C] 120/30  [1] 19/6  [2] 19/6  [3] 22/3  [4] 19/6  [5] 20/5  [6] 21/4 | 4.2 [4]  4.6 [5]  4.0 [4]  4.1 [4]  3.9 [5]  4.6 [5]  4.1 [4] | +10.5 [0]  +24.3 [+25]  +2.6 [+33]  0 [-20]  -2.5 [+25]  +35.3 [+67]  +10.8 [0] | **0.0015**  **0.0418**  0.2608  0.6099  0.4922  **0.0009**  0.1525 |
| N2  *vit-6* RNAi | [C] 134/16  [1] 23/2  [2] 24/1  [3] 19/6  [4] 24/1  [5] 23/2  [6] 21/4 | 3.8 [4]  3.6 [4]  3.8 [4]  3.7 [4]  3.6 [3]  4.0 [4]  4.0 [4] | 0 [0]  -2.7 [0]  -2.6 [+33]  -9.8 [-20]  -10.0 [-25]  +17.6 [+33]  +8.1 [0] | 0.7467  0.9702  0.8654  0.7633  0.2778  0.0850  0.2492 |
| N2  *vit-5,-6* RNAi | [C] 137/13  [1] 24/1  [2] 19/6  [3] 22/3  [4] 22/3  [5] 25/0  [6] 25/0 | 3.7 [4]  3.5 [3]  3.8 [4]  3.9 [3]  3.6 [3]  3.5 [4]  3.7 [4] | -2.6 [0]  -5.4 [-25]  -2.6 [+33]  -4.9 [-40]  -10.0 [-25]  +2.9 [+33]  0 [0] | 0.6532  0.5657  0.3764  0.4492  0.5647  0.7570  0.9808 |

**Supplementary Table 2**: Summary statistics for survival analyses for N2 on *vit* RNAi with 40 mM paraquat (cf. Figure 5A)

^1^Numbers in parenthesis indicate individual trials. [C], combined (pooled) data from all trials.

**Supplementary Table 3**: Summary statistics for survival analyses for N2 on *vit* RNAi with

7.5 mM *t*-BOOH (cf. Figure 5B)

| **Strain/**  **conditions** | **Number of deaths/censored**^1^ | **Mean [median] survival (hr)** | **% change vs.**  **control** | ***p* vs. control**  **(log rank)** |
| --- | --- | --- | --- | --- |
| N2  L4440 (RNAi control) | [C] 142/8  [1] 47/3  [2] 48/2  [3] 47/3 | 14.1 [15]  14.7 [15]  14.3 [15]  13.3 [13] |  |  |
| N2  *vit-5* RNAi | [C] 142/8  [1] 48/2  [2] 46/4  [3] 48/2 | 15.3 [15]  15.6 [15]  16.1 [17]  14.3 [15] | +8.8 [0]  +6.2 [0]  +13.1 [+13]  +7.5 [+15] | **0.0029**  0.1596  **0.0061**  0.2485 |
| N2  *vit-6* RNAi | [C] 144/6  [1] 48/2  [2] 50/0  [3] 46/4 | 15.0 [15]  15.4 [15]  14.6 [15]  14.0 [15] | +6.2 [0]  +5.1 [0]  +2.6 [0]  +5.6 [+15] | 0.1400  0.2777  0.4849  0.4419 |
| N2  *vit-5,-6* RNAi | [C] 146/4  [1] 49/1  [2] 48/2  [3] 49/1 | 14.2 [15]  14.4 [15]  14.3 [13]  13.9 [15] | +0.8 [0]  -1.6 [0]  0 [-13]  +4.3 [+15] | 0.7076  0.9685  0.9777  0.4572 |

^1^Numbers in parenthesis indicate individual trials. [C], combined (pooled) data from all trials.

**Supplementary Table 4**. Comparative abundance of *vit* mRNAs in RNA-seq and ribo-seq profiles. Whole worm mRNA, 1 day old hermaphrodites; data from Arnold *et al*. (2014).

| RNA-seq abundance ranking | Gene name | mRNA-seq, RPKM^1^ | Ribo-seq abundance ranking | Ribo-seq, RPKM^1^ |
| --- | --- | --- | --- | --- |
| 1 | *his-64* | 24104 | NA | NA |
| 2 | *ctc-3* | 7461 | NA | NA |
| 3 | *vit-6* | 4981 | 6 | 5932 |
| 4 | *rps-0* | 4909 | 17 | 4351 |
| 5 | *col-124* | 4673 | 11 | 5161 |
| 6 | *ctc-1* | 4384 | NA | NA |
| 7 | *dct-16* | 4099 | 75 | 2338 |
| 8 | *rpl-21* | 3804 | 53 | 2914 |
| 9 | *eef-1A.1* | 3757 | 2 | 10963 |
| 10 | *his-60* | 3756 | NA | NA |
| 11 | *his-57* | 3637 | NA | NA |
| 12 | *col-119* | 3409 | 8 | 5552 |
| 13 | *ctb-1* | 3387 | NA | NA |
| 14 | *rps-26* | 3348 | 73 | 2420 |
| 15 | *rpl-2* | 3323 | 146 | 1103 |
| 16 | *rps-2* | 2852 | 119 | 1405 |
| 17 | *rpl-17* | 2812 | 116 | 1585 |
| 18 | *col-181* | 2790 | 13 | 5042 |
| 19 | *rps-8* | 2773 | 32 | 3384 |
| 20 | *col-140* | 2767 | 3 | 7482 |
| 21 | *far-2* | 2658 | 10 | 5191 |
| 22 | *rps-23* | 2644 | 35 | 3309 |
| 23 | *his-66* | 2626 | NA | NA |
| 24 | *col-20* | 2610 | 5 | 6166 |
| 25 | *rps-7* | 2582 | 1 | 17836 |
| 26 | *rps-24* | 2572 | 20 | 4041 |
| 27 | *vit-5* | 2545 | 141 | 1181 |
| 28 | *col-81* | 2534 | 12 | 5077 |
| 29 | *tct-1* | 2343 | 101 | 1749 |
| 30 | *rps-12* | 2338 | 90 | 2075 |
| 31 | *rpl-15* | 2311 | 40 | 3234 |
| 32 | *rla-1* | 2215 | 56 | 2791 |
| 33 | *rpl-43* | 2213 | 33 | 3369 |
| 34 | *rpl-24.1* | 2211 | 38 | 3246 |
| 35 | *rpl-16* | 2187 | 85 | 2158 |
| 36 | *col-106* | 2185 | 47 | 3089 |
| 37 | *rla-0* | 2168 | 68 | 2558 |
| 38 | *atp-6* | 2162 | NA | NA |
| 39 | *col-139* | 2155 | 16 | 4440 |
| 40 | *rps-19* | 2142 | 58 | 2761 |
| 41 | *vit-2* | 2117 | 42 | 3231 |
| 42 | *rpl-36.A* | 2117 | 93 | 1958 |
| 43 | *col-129* | 2099 | 7 | 5719 |
| 44 | *ctc-2* | 2026 | NA | NA |
| 45 | *rpl-12* | 2022 | 87 | 2120 |
| 46 | *rps-1* | 2020 | 96 | 1808 |
| 47 | *rpl-26* | 1957 | 86 | 2141 |
| 48 | *rpl-11.1* | 1949 | 22 | 3933 |
| 49 | *vit-3* | 1910 | NA | NA |
| 50 | *rpl-27* | 1905 | 37 | 3247 |
| 51 | *vit-4* | 1864 | NA | NA |

^1^RPKM, Reads Per Kilobase Million.

**Supplementary Table 5**. Comparative abundance of *vit* mRNAs in RNA-seq and ribo-seq profiles. Whole worm mRNA, 1 day old hermaphrodites; data from Arnold *et al*. (2014).

| RNA-seq abundance ranking | Gene name | mRNA-seq, RPKM^1^ | Ribo-seq abundance ranking | Ribo-seq, RPKM^1^ | Ribo-seq/RNA-seq RPKM^1^ |
| --- | --- | --- | --- | --- | --- |
| 4 | *rps-0* | 4909 | 17 | 4351 | 0.9 |
| 5 | *col-124* | 4673 | 11 | 5161 | 1.1 |
| 7 | *dct-16* | 4099 | 75 | 2338 | 0.6 |
| 8 | *rpl-21* | 3804 | 53 | 2914 | 0.8 |
| 9 | *eef-1A.1* | 3757 | 2 | 10963 | 2.9 |
| 12 | *col-119* | 3409 | 8 | 5552 | 1.6 |
| 14 | *rps-26* | 3348 | 73 | 2420 | 0.7 |
| 15 | *rpl-2* | 3323 | 146 | 1103 | 0.3 |
| 16 | *rps-2* | 2852 | 119 | 1405 | 0.49 |
| 17 | *rpl-17* | 2812 | 116 | 1585 | 0.6 |
| 18 | *col-181* | 2790 | 13 | 5042 | 1.8 |
| 19 | *rps-8* | 2773 | 32 | 3384 | 1.2 |
| 20 | *col-140* | 2767 | 3 | 7482 | 2.7 |
| 21 | *far-2* | 2657 | 10 | 5190 | 2.0 |
| 22 | *rps-23* | 2643 | 35 | 3309 | 1.3 |
| 24 | *col-20* | 2609 | 5 | 6165 | 2.4 |
| 25 | *rps-7* | 2581 | 1 | 17836 | 6.9 |
| 26 | *rps-24* | 2571 | 20 | 4040 | 1.6 |
| 28 | *col-81* | 2534 | 12 | 5077 | 2.0 |
| 29 | *tct-1* | 2343 | 101 | 1749 | 0.7 |
|  |  |  |  |  | **Mean: 1.63** |
| 3 | *vit-6* | 4981 | 6 | 5932 | 1.2 |
| 27 | *vit-5* | 2544 | 141 | 1180 | 0.5 |
| 41 | *vit-2* | 2117 | 42 | 3231 | 1.5 |
| 49 | *vit-3* | 1910 | NA | NA | - |
| 51 | *vit-4* | 1864 | NA | NA | - |
| 102 | *vit-1* | 1193 | 109 | 1651 | 1.4 |
|  |  |  |  |  | **Mean: 1.15** |

^1^RPKM, Reads Per Kilobase Million.
